# Modeling Reveals Bistability and Low-Pass Filtering in the Network Module Determining Blood Stem Cell Fate

**DOI:** 10.1371/journal.pcbi.1000771

**Published:** 2010-05-06

**Authors:** Jatin Narula, Aileen M. Smith, Berthold Gottgens, Oleg A. Igoshin

**Affiliations:** 1 Department of Bioengineering, Rice University, Houston, Texas, United States of America; 2 Cambridge Institute for Medical Research, University of Cambridge, Cambridge, United Kingdom; California Institute of Technology, United States of America

## Abstract

Combinatorial regulation of gene expression is ubiquitous in eukaryotes with multiple inputs converging on regulatory control elements. The dynamic properties of these elements determine the functionality of genetic networks regulating differentiation and development. Here we propose a method to quantitatively characterize the regulatory output of distant enhancers with a biophysical approach that recursively determines free energies of protein-protein and protein-DNA interactions from experimental analysis of transcriptional reporter libraries. We apply this method to model the Scl-Gata2-Fli1 triad—a network module important for cell fate specification of hematopoietic stem cells. We show that this triad module is inherently bistable with irreversible transitions in response to physiologically relevant signals such as Notch, Bmp4 and Gata1 and we use the model to predict the sensitivity of the network to mutations. We also show that the triad acts as a low-pass filter by switching between steady states only in response to signals that persist for longer than a minimum duration threshold. We have found that the auto-regulation loops connecting the slow-degrading Scl to Gata2 and Fli1 are crucial for this low-pass filtering property. Taken together our analysis not only reveals new insights into hematopoietic stem cell regulatory network functionality but also provides a novel and widely applicable strategy to incorporate experimental measurements into dynamical network models.

## Author Summary

Hematopoiesis—blood cell development—has long served as a model for study of cellular differentiation and its control by underlying gene regulatory networks. The Scl-Gata2-Fli1 triad is a network module essential for the development of hematopoietic stem cells but its mechanistic role is not well understood. The transcription factors Scl, Gata2 and Fli1 act in combination to upregulate transcription of each other via distal enhancer site binding. Similar network architectures are essential in other multipotent cell lines. We propose a method that uses experimental results to circumvent the difficulties of mathematically modeling the combinatorial regulation of this triad module. Using this dynamical model we show that the triad exhibits robust bistable behavior. Environmental signals can irreversibly switch the triad between stable states in a manner that reflects the unidirectional switching in the formation and subsequent differentiation of hematopoietic stem cells. We also show that the triad makes reliable decisions in noisy environments by only switching in response to transient signals that persist longer than the threshold duration. These results suggest that the Scl-Gata2-Fli1 module possibly functions as a control switch for hematopoietic stem cell development. The proposed method can be extended for quantitative characterization of other combinatorial gene regulatory modules.

## Introduction

Appropriate spatiotemporal control of gene expression is central to metazoan development. [1]. Combinatorial interactions of regulatory proteins with regulatory regions of DNA and the basal transcriptional machinery form the building blocks of complex gene regulatory networks (GRNs). The availability of whole genome sequences as well as advanced bioinformatics and high-throughput experimental techniques have vastly accelerated the identification of candidate regulatory sequences. However, experiments that can uncover and/or validate the underlying connectivity of GRNs remain both costly and time consuming. Consequently, our understanding of the functionality of GRNs even for the most studied model organisms remains superficial. Moreover, simply cataloguing ever increasing numbers of interactions between GRN components is not sufficient to deduce the underlying network architecture or function of individual modules.

Unraveling the dynamical properties of GRNs will be the key to understanding their functionality. Throughout development, cells progress through a succession of differentiation steps from stem cells via immature progenitors to fully differentiated mature cells, and each of these subtypes is associated with a unique regulatory state of the GRN [1]. It is therefore essential to understand dynamical properties of the various regulatory states of GRNs, transitions between them and their interplay with intercellular signaling. It is unlikely that this goal can be achieved solely using experimental approaches. However, the development of dynamical models of GRNs offers great potential to interpret existing experimental data in order to gain new mechanistic insights.

Various computational approaches have been used for regulatory network analysis in the past. Boolean models provide qualitative information about network behavior such as the existence of steady states and network robustness and are most useful for large networks or when experimental information is scarce [2], [3]. However to examine dynamical aspects, continuous ordinary differential equation (ODE) models are more appropriate. These models can be constructed with phenomenological descriptions of gene regulation in the form of Hill functions or based on more detailed biophysical mechanisms and derived using a statistical thermodynamics approach. Phenomenological models are useful for understanding the general dynamics of network topology. They are most effective for small to medium sized networks and can also be predictive of cellular behavior [4]. Models based on thermodynamics have the advantage of including an hypothesis about the biophysics of the system [5], [6], [7]. Most parameters in these models have a direct biochemical interpretation. Unfortunately the lack of knowledge about specific biochemical parameters usually makes it difficult to relate results from these models to experimental information about gene expression. Nevertheless this modeling approach has been shown to be useful in understanding certain bacterial gene regulation modules [8] and studying the effects of nucleosome dynamics in eukaryotic gene regulation [9].

**Figure 1:**
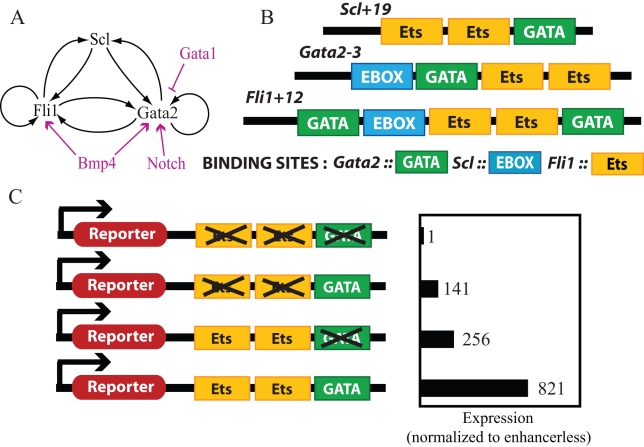
Regulation of gene expression in the Scl-Gata2-Fli1 triad. **A**. Scl, Gata2 and Fli1 form a triad module of TRs in the GRN of hematopoietic stem cells. The triad architecture consists of multiple positive feedback loops. Signals activating or deactivating the network are shown in magenta. Notch activates the transcription of Gata2 and Bmp4 activates the transcription of Gata2 and Fli1 by acting at the promoters. Gata1 binds to the Gata2 enhancer and downregulates Gata2 expression. **B**. The triad proteins regulate each other's transcription by acting at the *Scl+19*, *Gata2-3* and *Fli1+12* enhancers. These enhancers contain multiple binding sites that allow combinatorial control of gene expression. Only sites significantly affecting expression are shown **C**. Enhancer libraries similar to the one shown for Scl were constructed for all three proteins and subcloned with a suitable reporter in and in triad expressing cells to characterize the combinatorial control of gene expression. Typical results show the enhancement of gene expression from TR binding sites individually and in combination relative to enhancerless expression of the reporter.

The hematopoietic system has long served as a powerful model to study the specification and subsequent differentiation of stem cells [10]. Sophisticated cell purification protocols coupled with powerful functional assays have allowed a very detailed reconstruction of the differentiation pathways leading from early mesoderm via hemangioblasts and hematopoietic stem cells (HSCs) to the multiple mature hematopoietic lineages. Transcriptional regulators (TRs) have long been recognized as key hematopoietic regulators but the wider networks within which they operate remain ill defined [11]. Detailed molecular characterization of regulatory elements (enhancers/promoters) active during the early stages of HSC development has identified specific connections between major regulators [12], [13], [14], [15] and has led to the definition of combinatorial regulatory codes specific for HSC enhancers [16], [17], [18]. Moreover, these studies identified a substantial degree of cross-talk and positive feedback in the connectivity of major HSC TRs [19]. In particular, a triad of HSC TRs (Gata2, Fli1, Scl/Tal1) forms a regulatory module that appears to lie at the core of the HSC GRN [20]. This module consists of the three transcription factor proteins as well as three regulatory elements through which they are connected via cross-regulatory and autoregulatory interactions [12], [20] (Figure 1A). The details of regulatory interactions in this triad are shown in Figure 1B; only significant binding sites in the enhancers are shown for simplicity. *Gata2-3* and *Fli1+12* enhancers contain multiple Gata2 (GATA), Fli1 (ETS) and Scl (E-BOX) binding motifs. The *Scl+19* enhancer contains ETS and GATA binding motifs. Scl, Gata2 and Fli1 are all essential for normal hematopoiesis in mice [12] suggesting that the triad is an important sub-circuit or kernel of the GRN that governs hematopoiesis.

The triad architecture (Figure 1A) is very dense in regulatory connections and possesses multiple direct and indirect positive feedback loops. Such network topologies are rare in prokaryotes [21] but have been identified in other stem cell systems such as the Nanog-Oct4-Sox2 triad in the embryonic stem cell GRN [22], [23]. These observations suggest that the triad design may be associated with stem cell behavior. This idea prompted further investigation of combinatorial control by the triad TRs [20]. Generation of an enhancer library with wild type and mutant enhancers allowed the construction of different combinations of binding motifs in each enhancer. Wild type and mutant enhancers were sub-cloned into a SV minimal promoter and *lacZ* reporter vector and tested using stable transfection of hematopoietic progenitor cell lines [20]. This analysis produced results such as those schematically illustrated in Figure 1C.

It has been suggested that the dense connectivity and positive feedback loops within stem cell GRN modules play important roles in stabilizing the stem cell phenotype [20]. However, the dynamical nature as to how this self-enforcing circuit may be initiated or indeed exited remains unclear. In this paper we construct a mathematical model of the Scl-Gata2-Fli1 triad module and characterize its dynamical properties using continuous ODE modeling approaches. We first propose a thermodynamic method of estimating free energies of different configurations of the enhancer regions from the measurements of the transcriptional reporter libraries. This method together with a proposed biochemical mechanism of distant transcriptional enhancement significantly reduces dimensionality of the network parameter space. Measurements of protein lifetimes provide experimentally informed timescales to model transient behavior of the network. We analyze the network response to physiologically relevant signals such as Notch, Bmp4 and Gata1 and show that the network behaves as an irreversible bistable switch in response to these signals. Our model also predicts the results of various mutations in the enhancer sequences and shows that the triad module can ignore transient differentiation signals shorter than threshold duration. The combination of a bistable switch with short signal filtering not only provides new mechanistic insights as to how the Scl-Gata2-Fli1 triad may function to control HSC specification and differentiation but also suggests a possibly more general role for this network architecture in the development of other major organ systems.

## Results

### Thermodynamic model for enhancement of gene expression

Full quantitative characterization of the combinatorial nature of transcriptional regulation requires measurements of binding affinities between the DNA and TRs as well as interaction strengths among TRs. Moreover, the contribution of each individual TR and each possible combination to the transcriptional rate must be assessed. This information is extremely tedious to measure due to the combinatorial multiplicity of TR configurations and does not exist for the majority of experimental systems. Experimental data for synthetic libraries of transcriptional reporters that contain the gene regulatory elements is more readily available. We develop thermodynamic methods to characterize the combinatorial transcriptional regulation by distal enhancers based on this type of data and apply it to model the Scl-Gata2-Fli1 triad - a core module of the GRN of hematopoietic stem cells. Recently this system has been experimentally characterized [20]. In this study distal enhancer regions regulating the transcriptional rate of network proteins were identified and the relative contributions of each of the regulatory motifs were thereafter assessed individually and in combination by the use of a suitable transcriptional reporter (e.g., luciferase, lacZ). The typical results from these experiments are illustrated in Figure 1; see Table S1 for the full data used. We use this data to obtain the functional form describing the transcriptional rate of the reporter-enhancer constructs and estimate the biochemical parameters characterizing this function. Below we illustrate our approach for the *Scl+19* enhancer; the full model is derived in the methods section.

We assume that the distant enhancers increase the transcriptional rate via modulation of chromatin remodeling rather than through direct interaction with transcriptional machinery. This assumption is motivated by the observations that activation of the *Scl+19* enhancer is only revealed upon integration of the enhancer-promoter construct into chromatin and that the activity of the enhancer is independent of its position (upstream or downstream) relative to the reporter gene [20], [24]. Moreover, when integrated as single copy reporters into the genome of embryonic stem cells and assayed following 5 days of in vitro differentiation, the difference between wild type and mutant enhancer constructs lies in the number of cells that express the transgene rather than the level at which it is expressed (cf. Figure S1 and Text S1). Taken together, these observations suggest that chromatin dynamics play a significant role in the action of TRs at the enhancers. In the absence of enhancer binding, the gene can be in either open or a relatively stable closed chromatin state. In the closed chromatin state the binding regions for the TRs and the transcriptional machinery are wrapped in nucleosomes and are inaccessible; thus no gene expression is possible from this state. The closed chromatin state can spontaneously unwrap to an open state where the binding sites become accessible to allow polymerase to bind to the promoter and initiate transcription. Since most promoters bind RNA polymerase weakly, the probability of RNA polymerase binding and subsequently transcription rate 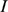 is proportional to the probability of the chromatin being in the open state (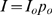; see Methods Eqs (15)–(17)). This probability depends on the equilibrium between open and closed chromatin states. Binding of the TRs at the enhancer stabilizes the open conformation thus shifting the equilibrium towards the open state (cf. Figure S2). This way the probability of open conformation increases with increase in TR concentration or increase in binding affinity. The rate of gene expression is still given by 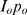 but 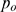 is now defined by a more complicated thermodynamic expression accounting for all the possible configurations of TR binding. Mutations in the enhancer site eliminate the configuration of TR binding thereby affecting 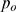 but not 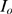. Below we illustrate this formalism for the *Scl+19* enhancer.

The *Scl+19* enhancer contains binding sites for Gata2 and a Fli1 dimer and therefore can exist in closed and four different open states (enhancer empty, Gata2 bound, Fli1 dimer bound, both Gata2 and Fli1 bound).The cumulative probability of all open state configurations is then given by 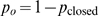, where 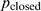 is the probability of the closed state given by
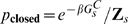
(1)where subscript *s* denotes the the *Scl+19* enhancer: 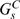 is the effective closed state energy, and 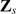 is the partition function given by the sum of exponentiated free energies 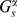 of each state 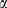: 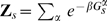. 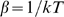 is an inverse temperature and hereafter all free energies are in its units. For TR-bound states, free energies are concentration dependent due to the loss of entropic degrees of freedom, *e.g.* for the Gata2-bound state 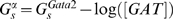, where 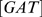 denotes concentration of Gata2. (Similarly 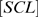 and 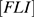 denote concentrations of Scl and Fli1 respectively). Since the free energies are only defined up-to a constant we can choose the free energy of the open state to be zero and thus obtain the following expression for the partition function:
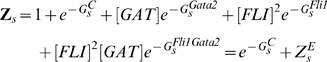
(2)where 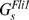 and 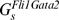 represent the free energies of Fli1 dimer and Gata2-Fli1 multimer binding and 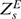 is the partition function for all open chromatin states. We use the subscript 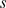 in all these terms to specify that they are associated with the *Scl+19* enhancer and the superscript to specify the binding configuration (cf. Table S2 for notation).

Direct measurements of the binding free energies in this expression may be tedious but these can be straightforwardly computed from the ratios of the transcription rates from synthetic reporter libraries with full or mutated enhancer sites. Ratios of the reporter expression levels of cell lines with wild-type (wt) and mutated (mut) enhancers can be used as constraints on the values of the binding free energies.
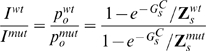
(3)Equations similar to (3) can be constructed for all reporter-enhancer libraries and used to recursively compute the binding free energies (cf. Eqs (22)–(27) in Methods and Eqs (S.1)–(S.11) in Text S3).

### Mathematical model for the Scl-Gata2-Fli1 Network triad module

Scl, Gata2 and Fli1 form an interconnected triad of positive interactions and play an important role in hematopoietic differentiation [12], [20]. To understand the role of the unique architecture of the triad module we construct a dynamical model of the system.

Assuming first-order degradation kinetics, deterministic rate equations for the change in TR concentrations take the form
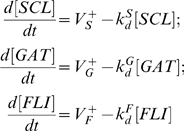
(4)where the functions 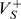, 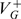 and 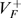 describes the rates production whereas 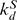, 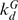 and 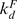 denote degradation rate constants for Scl, Gata2 and Fli1 respectively. Rate constants for protein degradation are estimated from known half-lives of the proteins. Since proteins are long-lived relative to mRNA, we can assume that production rates are directly proportional to the respective transcription rates 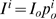 (cf. Eq (28)).

In addition to distant enhancers, Notch and Bmp4 are known to serve as activators of the promoters of Gata2 and Fli1, Gata2 respectively [25], [26]. These activators increase the rate of transcription by increasing the recruitment of RNA polymerase to the respective promoter. In particular, Notch and Bmp4 increase Gata2 expression by 3.5 fold [26] and 4 fold [27] respectively. In this case, to compute 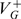 one needs thermodynamic expressions of the probabilities of multiple open conformations corresponding to binding of Notch or Bmp4. These probabilities depend upon Notch and Bmp4 concentrations (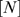 and 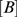 respectively) and their binding energies 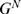 and 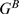 via the full partition function 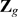 (subscript g stands for *Gata2-3* enhancer):
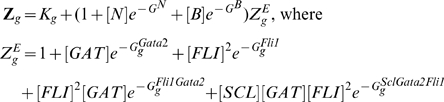
(5)Here 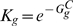 is the equilibrium constant for chromatin transitions between open and closed states for Gata2 enhancer (similarly 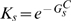 and 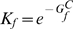 for *Scl+19* and *Fli1+12* enhancers respectively). These equilibrium constants are dimensionless quantities characterizing the maximum possible fold enhancement of gene expression by the respective enhancer. The partition functions are used to compute Gata2 synthesis rate 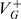 (cf. Eq (20)). The same procedure is used to describe the rate of expression of Fli1, although in this case only Bmp4 acts at the promoter (cf. Eqs (21)).

Conversion to dimensionless form can greatly simplify the model allowing easy interpretation of simulation results. We normalize the species concentrations of Scl, Gata2 and Fli1 as 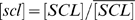, 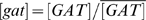 and 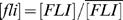. 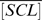, 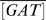 and 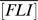 represent the mean observed concentrations of Scl, Gata2 and Fli1 in wildtype HSCs where the triad is actively expressed. In addition, 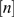 and 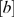 are Notch and Bmp4 concentrations normalized with respect to their promoter dissociation constants. With these normalizations, wildtype HSCs in the absence of signals would have 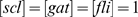 and 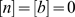. We choose this state as a reference state for the estimation of free-energies (cf. Methods Section for details). The dimensionless form of equation (4) is then given by
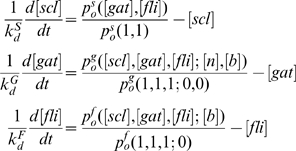
(6)Where 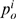 are dimensionless synthesis rates (cf. Eq 25). Note that in the final form of our model equations the wild-type state of HSCs 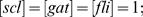 is always a steady state in the absence of signal 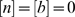. By using the parameter estimation method described in the previous section and reduction of the system to dimensionless form, we have reduced the dimensions of the parameter space and the only free parameters are the equilibrium constants for chromatin opening-closing 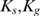 and 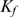. In the following sections we use this ODE model to analyze steady state and dynamical properties of this triad module.

### Steady state response of the triad module

**Figure 2:**
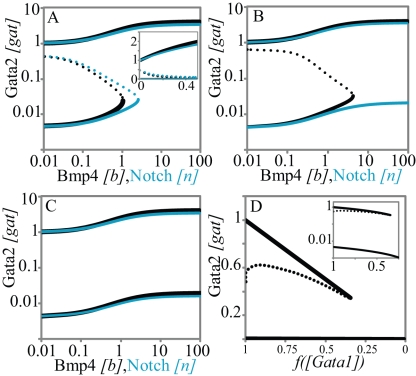
Steady state signal-response analysis of the triad module to Notch, Bmp4 and Gata1 signals demonstrates irreversible bistability. **A**. The action of Notch and Bmp4 at the promoters of switches the triad module from a low expression (*OFF*) state to a high expression (*ON*) state. Only Gata2 concentrations are shown for brevity. Solid lines represent stable and dotted lines represent unstable steady states. (Notch and Bmp4 concentrations are normalized by their respective binding affinities). Once the triad is in the *ON* state, the positive feedback loops in the modules architecture ensure that it remains in that state without signals (inset: the same plot in the linear scale). The switchability of the triad steady state response is sensitive to the values of 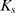 and 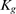. In **B** and **C**, we use different values for these chromatin equilibrium constants and recalculate all free energy values using the analytical equations derived with experimental results. For 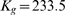 in **B**, only Bmp4 can switch the triad from *OFF* to *ON*. For 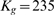 (C) neither Notch nor Bmp4 can switch the triad to *ON* state. **D**. Bistable response of the triad module to Gata1 repressor signal. Gata1 competes with Gata2 for binding sites on the *Gata2-3* enhancer and can switch the triad from *ON* state to *OFF* by decreasing the recruitment of RNA polymerase to the Gata2 promoter by a factor 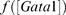. As a result the system irreversibly switches from *ON* to *OFF*. (note that this figure is shown in linear scale, the inset shows the deactivation in log-log scale for comparison with **A**). To evaluate the steady state dose response of each signal individually the concentrations of other signals were kept fixed at zero during simulation.

We use the model developed in the preceding sections to analyze the steady state response of the triad to Notch and Bmp4. By varying 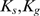 and 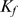 and calculating free energies that conform to the experimental predictions of mutant enhancer expression rates we can explore all regions of the relevant parameter space. Bifurcation analysis of the steady state response shows that the triad module has two stable steady states (see Figure 2). For certain values of the chromatin equilibrium constants Notch and Bmp4 can switch the triad between a low expression *OFF* state and a high expression *ON* state (Figure 2A). This switch in expression levels is irreversible and sustained even without Notch and Bmp4 signals. Therefore transient Notch/Bmp4 signals may lock the triad into the *ON* state. This irreversible progression switch behavior is expected from the triad module which has been reported to play a significant role in the specification of HSCs in the hemogenic endothelium. We use the above-described approaches to estimate the parameters for our model. Equations (25)–(27) relate the gene expression results from the *Scl+19* enhancer to the chromatin equilibrium constant 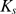. When we use these equations to estimate the free energies 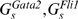 and 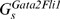 the model results match the experimental results exactly. The matching is only possible for the values of equilibrium constant above a threshold: 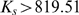. This lower bound is simple a consequence of the fact that in the proposed thermodynamic framework the maximal possible enhancement is given by 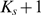 and the experimentally measurable enhancement is 820.51. Similarly the free energies for the *Gata2-3* and *Fli1+12* enhancers are estimated based on the experimental results and 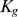 and 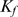 respectively (cf. Methods section and Text S3 for details). The values of these constants are also limited from below by the respective maximal measured enhancer factors.

In addition qualitative information about system behavior, namely its switchability as a response to physiologically relevant Notch and Bmp4 signals, places an upper bound on chromatin equilibrium constants values. For a different set of 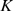 values the computed free energies are such that Notch and/or Bmp4 cannot cause the switch between low and high steady states (Figure 2B, C). As a result the system remains switchable in the very narrow range of two equilibrium constants 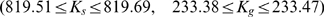 where the full enhancer brings the transcriptional rate to a nearly saturated value. The resulting narrow ranges do not indicate lack of model robustness but rather are a consequence of strict constraints placed on free energy values by the exact matching to the experimental reporter data (cf. equations (S.1)–(S.11) in Text S3). In fact without these constraints the range of 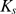 and 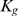 for switchable bistable response extends over several orders of magnitude (cf. Figure S3 and below). If we tolerate some deviation from the experimentally measured transcriptional data we can relax these constraints and significantly enhance the range of parameter values for which the system is bistable and switchable. For example, if we allow up to 20% deviation from transcriptional reporter measurements then the values of chromatin equilibrium constants can vary by 20% and still result in switchable response (data not shown). It is quite reasonable to tolerate such levels of deviation from the experimental results because the experimental results usually have a margin of error. Therefore we find that the qualitative predictions of the model (switchable bistable response) are robust however the quantitative predictions (transcriptional data) are only as accurate as the experimental data one which the model is based.

We expect the triad to be switchable in response to both Notch and Bmp4. Therefore we choose the chromatin equilibrium constants from within the narrow ranges shown above and calculate the TR-enhancer binding free energies using these chosen values. For this chosen set of parameter values the model shows an irreversible bistable response to Notch and Bmp4 (Figure 2A). Bmp4 concentrations were set to zero for evaluating the Notch dose response and vice versa. The presence of one signal reduces the threshold concentration of the other signal at which the triad switches from *OFF* to *ON* (data not shown). The calculated free energies are shown in Table S3 and used through the remaining simulations. Once the free energies of TR binding are fixed at Table S1 values, the system becomes robust to variability of chromatin equilibrium constants (Figure S3). Such changes may biologically correspond to histone modification or other physical perturbations. In response to changes over a large range the triad shows switchable and irreversible bistable responses to Notch and Bmp4 (Figure S3). Therefore the switchable nature of triad bistability is robust to several fold parameter changes.

Gata1 can displace Gata2 from its binding sites in the *Gata2-3* enhancer. Through competition for binding sites and subsequent chromatin remodeling Gata1 can switch the triad from high expression back to the low expression state. We represent the chromatin remodeling effect of Gata1 by including a factor 0<
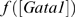
<1 in our expression for the rate of Gata2 gene transcription 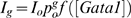. Because the exact biochemical mechanism of the Gata1 action is not established we choose a decreasing function of Gata1 and make no other assumptions about the functional form of 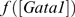. We therefore, plot Gata1 dose-response curves with 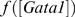 as the x-axis where its values decrease left to right (Figure 2D). This phenomenological description of the effect of Gata1 captures the effect it has on RNA polymerase recruitment to the promoter by initiating chromatin remodeling. Inclusion of Gata1 in our model (Figure 2D) allows the system to switch from *ON* to *OFF* states. The switching is irreversible – the system will remain *OFF* even after Gata1 signal is gone (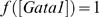). Notch and Bmp4 concentrations were fixed at zero for evaluating the Gata1 response because the concurrence of Notch/Bmp4 and Gata1 signals is physiologically unlikely.

Interestingly, Gata1-deactivation is far more susceptible to noise than the activation by Notch/Bmp4. This can be concluded from the dotted line representing the unstable steady state that separates the stable *ON* and *OFF* states (compare Figures 2A and D). This line characterizes the magnitude of concentration fluctuations required for spontaneous transitions. For sub-threshold signals, this line is much closer to the stable steady state in Gata1 dose-response curves (Figure 2D) as compared to Notch or Bmp4 curves (Figure 2A). A more rigorous investigation of the magnitude of stochastic effects and their relation to separatrix of deterministic model requires a full stochastic model of the network and will be conducted elsewhere.

### Mutations in the enhancer sites change the steady-state response of the triad

We expect the steady state response of the Scl-Gata2-Fli1 module depends on the triad architecture and design of enhancers. The model presented above allows us to verify this claim by introducing changes in the triad design corresponding to mutations of enhancer sequence and gene knockouts and examining the effects on the steady state response. To this end, we systematically deleted TR-binding sites from each enhancer *in silico* and analyzed the steady state response of the system. We also analyze the steady state response of *Scl*, *Gata2* and *Fli1* deletion mutants.

**Figure 3:**
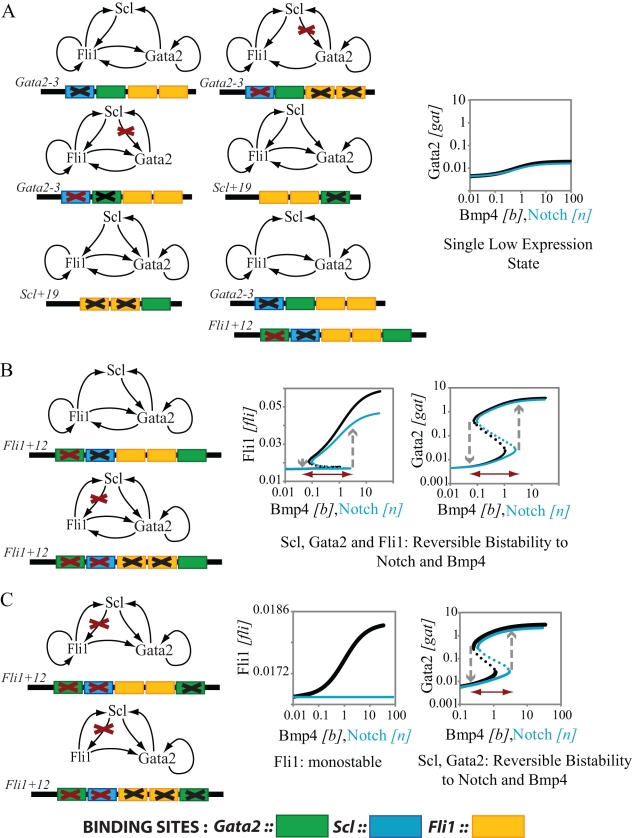
Selective deletion of enhancer binding sites can change the steady state response characteristics. **A**. Deletion of any of the enhancer binding sites from the *Scl+19* or *Gata2-3* enhancers eliminates the high expression state of Scl, Gata2 and Fli1 seen in the wildtype HSCs. Black crosses mark the deleted sites, red crosses mark the interactions that are no longer significant as a result of the deletion. **B**. Mutations in the Scl or Fli1 binding site in the *Fli1+12* enhancer allow triad activation but lead to reversible bistability-the *ON* state switches back to *OFF* in the absence of Notch and Bmp4. **C**. Deletion of the primary Gata2 binding site from the *Fli1+12* enhancer makes the Scl interaction with the enhancer insignificant. This effectively makes Fli1 independent of external regulators Scl and Gata2. Fli1 expression is low for these mutants and monostable. Notch has no effect on Fli1 concentration. Gata2 and Scl show reversible bistability in response to Notch and Bmp4 in these mutants.

Mutations in the triad enhancer sequences can produce many modules with simpler architecture as shown in Figure 3. Notably, since some TR-enhancer configurations do not make a significant contribution to the enhancer activity, removal of a single enhancer binding site might effectively eliminate multiple TR-enhancer interactions. For example, the effect of Scl on the Gata2 and Fli1 enhancers is only significant when both Gata2 and Fli1 are bound to the enhancer. Therefore the probability of Scl bound enhancer configurations for these enhancers is negligible for any motif where the Gata2 or Fli1 sites on these enhancers are deleted.

Keeping this in mind we analyze 10 different triad module designs that can be obtained by selective single and double mutations of enhancer binding sites. The model described above is suitably altered to predict the steady state response of these alternate designs. All relevant parameter values are taken from the full triad model. Of the 10 “mutant” designs, all 6 modules where the *Scl+19* or *Gata2-3* enhancers are mutated show only a single steady state with the expression of Scl, Gata2 and Fli1 comparable to the low expression state of the full triad (cf. Figure 3A). On the other hand, high levels of expression can still be observed in 4 modules with mutations in the *Fli1+12* enhancer (see Figures 3B and 3C). However, in contrast to wild-type (Figure 2A), this high level of expression cannot be maintained in the absence of Notch and Bmp4. Even when the E-BOX biding site for Scl is eliminated from the *Fli1+12* enhancer the system remains bistable for a range of signal. For the designs in which the GATA site in the *Fli1+12* enhancer is eliminated (Figure 3C) Fli1 expression is uncoupled from Gata2 and Scl and is monostable while the responses of Scl and Gata2 are still bistable. This is expected because Fli1 autoregulation is not strong enough to produce bistability.

Complementarily, we can also assess the effects from alterations of TRs rather than their binding sites. Simulations show that Scl^−/−^, Gata2^−/−^ and Fli1^−/−^ knockout mutants cannot support the high expression state of the triad. These mutants produce a phenotype similar to the enhancer mutations in Figure 3A. Comprehensive analysis of knockout mice has shown that hematopoiesis is severely impaired in all three deletion mutants [28], [29], [30], [31]. Our model suggests that the knockout of any of the triad proteins prevents the switch to *ON* state which is likely to affect the specification of HSCs during early embryonic development and therefore compromise the development of all mature blood cell types as seen experimentally. On the other hand, the irreversible bistability of triad response is preserved if we delete one chromosomal copy of any one of the three triad genes; however the heterozygotic mutants are expected to be more prone to differentiation (cf. Figure S4 and Text S2). This could explain why these mutants have reduced repopulation capacity [32], [33].

### Dynamical response of the triad module architecture

**Figure 4:**
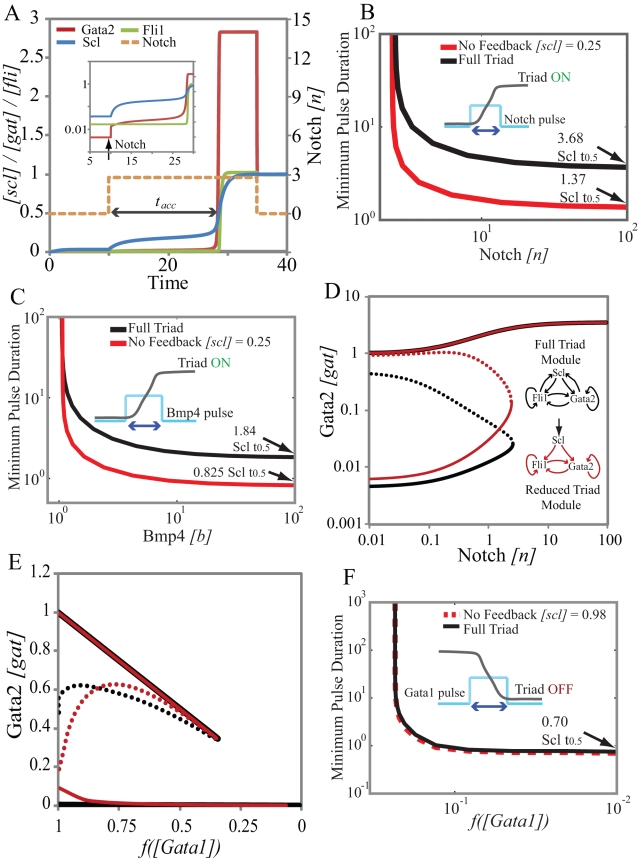
Comparison of dynamical responses of the triad and the reduced module to Notch, Bmp4 and Gata1 signals. **A**. A time course of the switching from low expression to high expression state in response to a pulse of Notch. The inset shows that there is an increase in Gata2 concentration immediately after the introduction of Notch. Scl starts accumulating slowly in response to this increase in Gata2 concentration but Fli1 concentration is stagnant because enough Scl is not present to appreciably increase Fli1 expression. Once Scl has reached the required concentration (t_acc_ after start of Notch pulse) Gata2 and Fli1 concentrations increase rapidly to the *ON* state level. Thus switching of the triad to the high expression *ON* state is rate-limited by the slow accumulation of Scl **B**. The minimum Notch pulse-duration required for *OFF*→*ON* switching as a function of pulse amplitude. Black line is the full triad the red curve is the reduced module with constitutive Scl (cf. text for details). **C**. Same as (**B**) but for Bmp signal. **D**. Steady-state response of the reduced module (red) with the Scl concentration fixed at the value that ensures that the switching threshold is identical to that of the wild-type triad (black). Note that the unsteady state (separatrix-dotted curves) for the reduced module is much closer to the *ON* state. **E**. Controlled comparison for deactivation by Gata1 steady-state response with Scl concentration fixed to ensure that the deactivation thresholds for both modules are identical. **F**. Transient filtering of Gata1 signals is very similar for the two designs since Scl does not limit the rate of response to Gata1.

The dynamics of the response of the bistable triad module to a pulse of Notch is illustrated in Figure 4A. The step increase in Notch concentration almost immediately increases Gata2 concentration slightly. However Fli1 concentration remains stagnant because Scl level rises very slowly. The slow speed of Scl response is governed by its slow degradation rate (half life ∼8 hrs). Once enough Scl has accumulated, the probability of Scl being present on the Gata2 and Fli1 enhancers becomes significant. This results in a rapid increase of expression rates and the triad switches to the high expression state. The rate limiting step for switching *ON* the triad expression levels is therefore the slow accumulation of Scl.

To further investigate the dynamics of triad switching in response to transient stimuli we have computed the minimal pulse duration that can cause irreversible switching as a function of signal amplitude (Figure 4 B, C; black lines). The results indicate that the system can be switched *ON* by signal pulses longer than a certain threshold level (∼42 hrs for a Notch pulse and ∼21 hrs for a Bmp4 pulse). This threshold is a few fold larger than Scl-lifetime, the longest timescale for the system. Our simulations therefore indicate that the triad module is capable of filtering transient signals that are shorter than the threshold simulation. We refer to this property as low-pass filtering – a term accepted for similar phenomena in engineering literature [34]. This filtering appears to be related to the slow turnover of Scl and the feedback loops connecting Scl with Gata2 and Fli1.

To understand how slow Scl dynamics contributes to the filtering of transient Notch and Bmp4 signals we compare the dynamics of the triad module to that of a simpler network module where the *Scl+19* enhancer has been deleted. We call this module the reduced module. In this reduced module Scl is assumed to be under an external regulator that controls Scl concentration. With this reduction, Scl concentration is constant and the dynamics of Gata2 and Fli1 response are not limited by the slow accumulation of Scl. For a controlled comparison of the dynamics [35] we assume that all relevant parameters have the same values as they do in the full triad model. This leaves the Scl concentration as the only free parameter. The reduced module shows irreversible bistable response to Notch, Bmp4 and Gata1 for a range of Scl values. We constrain the Scl concentration such that the threshold for *OFF* to *ON* transitions is the same for the reduced module and the full triad (Figure 4D). Notably, the separatrix between the two stable states (dotted line, Figure 4D) is much closer to the *ON* state for the reduced module. This suggests that the reduced module is more susceptible to fluctuations in TR levels as compared to the full triad.

We now use the reduced module as described above for a controlled comparison of the dynamics of the *OFF* to *ON* and *ON* to *OFF* switching. Both bistable switches act as filters for transient signals above the threshold (Figure 4 B and C). We compared this dynamic response of the triad and reduced modules to Notch and Bmp4 pulses. The models for the two modules have the same Notch/Bmp4 thresholds and close to the threshold the minimum pulse duration for both modules is high. However at higher concentrations of Notch and Bmp4, the minimum pulse duration is much higher for the triad module than for the reduced module (16 hrs and 9.5 hrs for Notch and Bmp4 pulses respectively). These results show how the slow dynamics of Scl allow the full triad module to act as a better low pass filter function for activation as compared to the reduced module.

For a controlled comparison of the response of the two modules to Gata1 we fix the Scl concentration of the reduced module such that the threshold level of Gata1 is identical (Figure 4E). This fixed concentration of Scl is 4 fold higher for deactivation than for activation. Gata1 acts at the *Gata2-3* enhancer to shut off transcription through chromatin remodeling. The slow dynamics of Scl do not affect the Gata2 concentration during this deactivation. As a result the deactivation dynamics and the minimum pulse duration for *ON* to *OFF* switching at high Gata1 concentrations (∼8 hrs) of the reduced module and the full triad are identical. The triad and reduced module are equivalent low pass filters for deactivation signals such as Gata1.

## Discussion

### A new method for determining free energies of TR-DNA interactions

Combinatorial gene regulation is ubiquitous in eukaryotes with complex DNA regulatory regions acting as integration points for multiple signals and pathways involved in gene regulation. The characterization of these regulatory regions through mathematical models is an important step towards understanding the functionality of gene regulatory networks. In order to fully characterize each regulatory element, one needs to determine dynamical functions that describe the rate of transcription as a function of TR concentrations. The most biochemically and biophysically realistic method of characterizing transcriptional regulation is rooted in statistical thermodynamics where each state of the regulatory region is assigned a free-energy so that the probability of each state can be computed from Boltzmann distribution [5]. These methods have been previously applied to bacterial systems [8] but rarely used for eukaryotic gene regulatory networks as a lack of reliable parameter measurements prevents researchers from undertaking detailed modeling approaches. Here we have developed a method for the quantitative characterization of combinatorial gene regulation by multiple TRs in eukaryotic distant enhancers. Our proposed method extends the thermodynamic approach of [36] in order to relate it to experimental transcriptional reporter assays. We develop a recursive method to estimate relevant free energies from the measurements of combinatorial libraries of transcriptional reporters. There are multiple benefits of computing free-energies of TR-DNA configurations. First, these parameters allow straightforward construction of mathematical models for quantitative analysis of system behavior with no or just a few free parameters. Second, free energies can be used for model reduction by specifically excluding thermodynamically unfavorable states and subsequent model reduction. Third, the parameters provide important qualitative insights into gene regulatory mechanisms such as cooperativity of TRs. We further reduce the number of parameters required to characterize the distant transcriptional enhancers by proposing a detailed mechanism based on the modulation of chromatin remodeling activity.

Chromatin structure is known to play an important role in eukaryotic gene regulation. The organization of DNA into nucleosomes can prevent the transcriptional machinery and regulatory factors from accessing regulatory regions. The detailed mechanism of action of distant enhancer sites has not been established. It has been suggested however that its action may involve modulation of chromatin remodeling dynamics [37]. For instance, regulatory elements of the Scl-Gata2-Fli1 triad were shown to be critically dependent on integration into chromatin [12]. Here we propose a ratchet mechanism of enhancer action (cf. Figure S2). We propose that DNA can be in a dynamic equilibrium between open (promoter site accessible) and closed (promoter site inaccessible) conformations. Such a dynamic equilibrium between wrapped and unwrapped nucleosomal DNA has also been discussed elsewhere [38]. In the absence of enhancer TRs, the equilibrium is heavily shifted towards a closed state resulting in very low transcription probability. We hypothesize that binding of TRs to the enhancer site stabilizes an open conformation and thereby shifts the equilibrium towards it. This mechanism therefore allows the TRs to ratchet the spontaneous unwrapping of nucleosomal DNA and trap it in a state accessible to the transcriptional machinery. We apply this thermodynamic framework to a regulatory module hypothesized to play a pivotal role in hematopoiesis. Under this assumption the binding of Fli1, Gata2 and Scl to their enhancer sites activates gene transcription by increasing the probability of transcription rather than the rate of transcription. This hypothesis is consistent with previously reported results of studies focused on enhancer function in mammalian cells [37], [39], [40] and with our flow cytometry experiments with cells containing the *Scl+19* enhancer-reporter constructs (cf. Figure S2 and Text S1).

The proposed mechanism assumes that the unwrapping of DNA from nucleosomes is independent of all triad factors and thus effectively spontaneous. However chromatin modification and chromatin remodeling factors can affect these nucleosome dynamics. In particular, factors such as the Gata2 repressor Gata1 may regulate the expression by modulating free energies of DNA unwrapping through chromatin modification. By shifting the equilibrium further towards the closed state, Gata1 can suppress transcription to such an extent that TR concentrations are too low to ratchet the very short-lived open state.

### Steady state characteristics of the Scl-Gata2-Fli1 triad

The recently characterized Scl-Fli1-Gata2 triad module includes a large number of transcriptional interactions resulting in multiple positive feedback loops. The complex enhancer structure makes it rather difficult to phenomenologically deduce dynamical expressions for Scl, Gata2 and Fli1 transcription. However, with our newly developed approach based on transcriptional reporter data, construction of a mathematical model of the triad becomes a straightforward task. The resulting model of the triad exhibited bistability in response to the action of Notch and Bmp4. We have chosen the free energy values for DNA unwrapping to ensure that the action of these two activators at the promoters switches the triad from low expression (*OFF*) state to high expression state (*ON*). The model predicts this switching to be irreversible – the triad will remain *ON* even after the signals are gone (Fig 2A). The development of HSCs in the hemogenic endothelium is known to be a Notch regulated event [41]. Notch is known to be expressed in endothelial cells and act as a regulator of Gata2 expression during the onset of hematopoiesis [26]. Bmp4 expression has also been observed in the dorsal aorta region where HSCs first develop in the embryo [18], [42]. Notch and Bmp4 are known to be mediators of HSC specification during embryonic development [41]. Our model shows how the action of Notch and Bmp4 is crucial for the *OFF* to *ON* switch of the Scl-Gata2-Fli1 triad. Since HSC specification requires Scl, our model predicts that in the absence of Notch and Bmp4, newly generated HSCs are trapped in a low expression state and hematopoietic development is compromised. The network also irreversibly switches from the *ON* to *OFF* state when reaching a threshold value of repression of Gata2 transcription by Gata1. The network will then remain in the *OFF* state in the absence of other signals. Interestingly, in ref. [22] the authors use a mathematical model to predict that a similar triad module in embryonic stem cells is also bistable. However their module is expected to be bistable only in the presence of activating or deactivating signals unlike Scl-Gata2-Fli1 triad that shows irreversible bistability.

Our analysis indicates essential roles of all the enhancer sites included in the model in maintaining irreversible bistability in steady state dose-response curves of the triad. Elimination of any binding sites in Scl or Gata2 enhancers leads to complete elimination of bistability with only the *OFF* state remaining. Mutations in the Fli1 enhancer may lead to a reversible bistability phenotype in which the triad is activated only in the presence of Notch and/or Bmp4 signals above a certain threshold. We emphasize, however, that these predictions do not indicate that simpler triad networks with less autoregulation are incapable of achieving irreversible bistable switching behavior. Our goal was to predict the behavior of the triad to the mutations of the regulatory regions. If one allows compensatory changes in other model parameters one can restore the irreversible switching behavior and even set the switching threshold to be equal to that of wild-type triad. However, as indicated below the reduced modules may still display physiologically important differences in other aspects of dynamic behavior.

### Transient responsiveness of the Scl-Gata2-Fli1 triad

In order to characterize the transient responses of the triad module one needs the values of kinetic parameters – lifetimes of triad proteins. Scl and Fli1 are known to be relatively stable proteins with measured half-lives of 8 hours and 2 hours respectively [43], [44]. Gata2 is comparatively unstable with a half-life less than 30 minutes [44]. This combination of short-lived and long-lived transcription regulators allows the triad to respond quickly to changes in mRNA transcription rates and at the same time, act as memory modules for history-dependent switches into and out of the HSC regulatory state. Analysis of the dynamical response of the triad to Notch/Bmp4 indicates that slow accumulation of Scl acts as a rate-limiting step for *OFF-ON* switching. As a consequence the triad must be exposed to Notch/Bmp4 signals for significant time periods for switching to occur. Physiologically this means that the triad motif works as a low-pass filter that responds only to transient stimuli longer than threshold duration and ignores brief, transient signals shorter than the threshold duration. All bistable switches show this type of threshold filtering of transient signals but to a different degree [45]. In our case, the response rate for the triad is limited by slow Scl dynamics and therefore multiple features of the triad network contribute to this property. For example, Scl is the slowest in degradation among the TRs and Notch/Bmp4 signals affect its accumulation only indirectly (Figure 1A). In addition, we hypothesized that the positive feedback loops involving Scl play a significant role in determining the threshold for low-pass filtering. We have confirmed this hypothesis by comparing the response of the triad to a hypothetical reduced module wherein the *Scl+19* enhancer is deleted and Scl acts as an external TR for the Gata2-Fli1 feedback loop [35]. We therefore conclude that the full triad is a better low pass filter because of the rate-limiting nature of Scl accumulation and Scl-mediated positive feedbacks significantly affect the signal filtering properties of the triad.

Studies in heterogeneous cell populations derived from differentiating ES cells or mouse fetal liver had suggested low level binding of Scl itself to the *Scl+19* enhancer [18]. However, more recent analysis in a clonal population of blood stem/progenitor cells did not detect any binding of Scl to this element [15]. Positive autoregulatory feedback through the *Scl+19* enhancer is therefore unlikely to play a significant role in stem cells, especially as the *Scl+19* element does not contain a bona fide binding site for Scl which would necessitate indirect binding. Nevertheless, we have considered the addition of a positive auto-feedback loop on Scl but simulations demonstrated that it does not generate a qualitatively different scenario with the only major consequence being a further slow-down of the switching rate due to the retardation of response by positive feedback (data not shown).

Gata1 acts at the *Gata2-3* enhancer and is reported to actively promote chromatin modification [46]. The decrease in Gata2 concentrations is not limited by Scl dynamics because Gata1 directly affects Gata2 transcription by reducing RNA polymerase recruitment. We therefore expected that filtering characteristics of the full and reduced triad motif would be the same. We performed a controlled comparison choosing a concentration of Scl in the reduced module (with the *Scl+19* deleted) that ensures the same switching threshold. The results indeed show essentially identical low-pass filtering properties of the two modules because Scl dynamics are not rate limiting in this case.

### The Scl-Gata2-Fli1 triad as a central regulator of stem cell fate

Experiments have shown that the knockout of any one of the genes Scl, Gata2 or Fli1 affects the development of HSCs and leads to severely impaired hematopoiesis. Thus the expression of these TRs is critical for hematopoiesis. More recent studies have shown that these three genes regulate each other by acting at distant enhancers as activators. Results from our model provide insight into the function of this module of TRs and suggest that the triad is a central regulator that controls the specification of HSCs during early hematopoiesis and the generation of progenitors committed to differentiation from these cells.

The bistable switch properties of the triad are hallmarks of a decision module. The triad switches irreversibly from the low to high expression state in response to external cues such as Notch and Bmp4 that are important for establishing definitive HSCs in the hemogenic endothelium. The bistable response predicted by the model is robust to fluctuations in parameter values. Experimental results also support this prediction [47]. The model shows that the knockout mutants are unable to reach the activated high expression state due to the all or none nature of this bistable response. Additionally the slow turnover of Scl retards the triad response to Notch and Bmp4 and thus makes it a highly effective low pass filter for noise in these signals.

The response to deactivation by Gata1 is not affected by Scl dynamics. As a result the *ON* to *OFF* switch for the triad is much faster than the *OFF* to *ON* switch. Deactivation by Gata1 is also more sensitive to stochastic fluctuations in triad protein concentrations. The cells can be switched to the *OFF* state to produce progenitor cells committed to differentiation by fluctuations in triad TR concentrations. Thus asymmetric partitioning of these proteins during cell division can allow sub-threshold Gata1 concentrations to silence Gata2 expression in one of the daughter cells by chromatin modification. The probability of this stochastic exit from the pluripotent HSC state of the cell is governed by the Gata1 concentration in the cell. This observation is consistent with experimental analysis of a multipotent hematopoietic progenitor cell line which demonstrated that these cells exist in two distinct subpopulations when cultured under self-renewal conditions with the more differentiation prone subpopulation expressing higher levels of Gata1 [48]. Of note, the triad switches between states in an all or none fashion where overexpression of exogenous Gata2 for example could prevent deactivation of the triad by Gata1. In line with these predictions, it has been demonstrated that overexpression of Gata2 in differentiating ES cells increases the production of hematopoietic progenitors and slows down their differentiation [49].

Our model of the triad module shows that it responds differently to activation and deactivation signals. This allows the *OFF* to *ON* and *ON* to *OFF* switches to fulfill different functional requirements. The activation response is slow, irreversible and robust to fluctuations in external signals to allow the development of HSCs in a noisy intercellular signaling environment. Simulation results for the dynamics of deactivation suggest that it may be faster than the *OFF* to *ON* switch and may exploit stochastic intracellular fluctuations during the cell cycle to maintain the HSC population and guarantee a continuous supply of lineage committed progenitors at the same time.

From a model based on the quantitative experimental characterization of the triad enhancers we have predicted several qualitative features of the steady state and transient response of the triad as well as its sensitivity to mutations and over-expression. We favored a deterministic model for our analysis of the triad function because of the reliability and robustness of the predictions that we have been able to extract from this approach. Even so, a stochastic model can potentially offer additional information about noise properties of the system and we intend to use results presented here to guide the construction of a full stochastic model in the future. Taken together the results presented here are consistent with prior experimental data and provide new mechanistic insights into potentially critical features of the regulatory networks that govern the specification and subsequent differentiation of hematopoietic stem cells. Moreover, our strategy of exploiting experimental data to infer biophysical properties should be widely applicable to aid regulatory network reconstruction in a wide range of cellular and developmental systems.

## Methods

### Modeling regulation at the enhancer level

We extend the Shea-Ackers [7] description of gene regulation to construct the deterministic models discussed above. The following assumptions are the foundation of this modeling approach,

1. The TR-DNA binding and unbinding processes are fast compared to transcription and translation and can be assumed to be at equilibrium. We note that the equilibrium assumption may only be applicable for the population-average deterministic model we construct here and may fail to accurately describe single-cell data.
2. The rate of gene transcription is linearly related to the probability of RNA polymerase (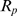) being bound to the promoter.

The assumption of equilibrium allows us to calculate the probability of finding TRs bound to DNA using the Boltzmann weighting factors for all configurations (occupied and unoccupied) of the DNA regulatory element [5]. The sum of the Boltzmann factors for all configurations is the partition function
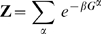
(7)Here 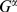 is the free energy of the state 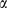 (we measure free energies 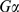 in units of 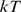 and use 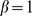). The partition function is used to calculate the probability of each of configuration. We distinguish three different types of regulatory element configurations based upon our model of nucleosome dynamics.

1. Closed chromatin configuration for the enhancers: The DNA of the regulatory enhancer is tightly wrapped around histones. No DNA binding proteins (including RNA polymerase) can access binding sites when the DNA is in this configuration. No gene transcription occurs while the gene is in closed chromatin state.
2. Open chromatin configurations: Spontaneous unwrapping of DNA from the histones produces a configuration where none of the TRs are bound to the enhancer but binding of RNA polymerase to the promoter is allowed. Gene transcription can happen in this state.
3. Occupied enhancer configurations: DNA is unwrapped from the histones and enhancers are occupied by TRs. This set of configurations includes all possible configurations of TRs at the enhancer. RNA polymerase can bind to the promoter in this state leading to gene transcription.

We use these definitions to formulate the probabilities 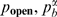 and 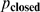 of open chromatin with no TR binding, different enhancer bound states 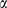 and closed chromatin respectively:
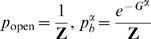
(8)

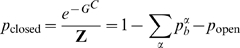
(9)Here the energy for all states is measured relative to the open chromatin state (which is set to zero).

The Gottgens group cloned the *Scl+19*, *Gata2-3* and *Fli1+12* enhancers upstream of a SV promoter controlling a *lacZ* reporter gene and integrated this construct into the genome of wild-type HSCs that show high expression of Scl, Gata2 and Fli1 [20]. In the presence of all three TRs, the enhancer can be occupied in many different TR configurations and reporter expression is significantly higher than constructs with no enhancer. Mutant enhancers where certain TR binding sites have been deleted were also used with reporter gene constructs to measure the gene expression enhancement. The results from these experiments show that only the deletion of certain critical enhancer binding sites affects gene expression enhancement. These critical sites are shown in Figure 1 and the experimental results from [20] are included in Table S1. We use these results to simplify the model of combinatorial gene regulation in the triad.

The expression of Scl is under the control of two TRs Gata2 and Fli1 with different binding sites in the *Scl+19* enhancer. The 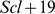 enhancer can therefore be in either closed state, open state, bound by Gata2, bound by Fli1 dimer or bound by Gata2 and Fli1 dimer simultaneously. Given the various configurations of the enhancer, the derivation of the partition function is straightforward (cf. Eq (2)).

We define 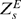 as the sum of the Boltzmann weights of all open state enhancer configurations for ease of representation of the probability of open chromatin states in equation (9). Note, that the binding energy 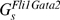 includes the TR-TR interaction of Gata2 and 2 Fli1 TRs while bound to DNA.

The *Gata2-3* enhancer includes binding sites for Gata2, Scl and the 2 Fli1 TRs. Many TR binding sites can be deleted without affecting the reporter gene expression enhancement [20]. The binding sites for Scl, Gata2 and Fli1 shown in Figure 1 are critical for gene expression enhancement. Gene expression is decreased but still significantly enhanced if only the Gata2 or Fli1 sites are present. Deletion of all sites except Scl binding site makes the expression enhancement negligible. However deletion of only the Scl site significantly decreases the expression enhancement from the full enhancer. These results suggest that although Scl binds weakly to the incomplete enhancer by itself, the Scl-Gata2-Fli1 complex has great affinity for the *Gata2-3* enhancer. Of all possible configurations of *Gata2-3* enhancer occupation only the Gata2 bound, Fli1 bound, Gata2-Fli1 bound and Scl-Gata2-Fli1 bound configurations are therefore included in the partition function 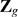 for *Gata2-3*.
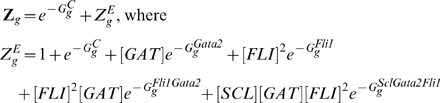
(10)
Figure 1 also shows the critical *Fli1+12* enhancer binding sites. This enhancer includes two Gata2 biding sites (primary site at *5′* end). The Scl binding site and the secondary Gata2 site (*3′* end) cannot enhance gene expression by themselves. The primary Gata2 site and the Fli1 dimer sites have some effect on gene expression and together they raise gene expression ∼20 fold. Single mutation of either the Scl or secondary Gata2 binding sites has a negligible effect on the gene expression. Deletion of both sites together reduces the gene expression enhancement from ∼60 fold to ∼20 fold. Thus the Gata2 bound, Fli1 bound, Gata2-Fli1 bound and Gata2-Scl-Fli1-Gata2 bound configurations have a significant effect on the gene expression. Incorporating these experimental results simplifies the partition functions 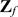 for Fli1.
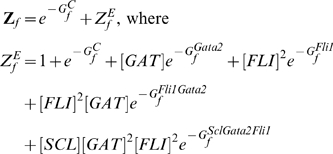
(11)


### Modeling regulation at the promoter level

So far we have enumerated all configurations of the enhancers. Notch (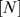), Bmp4 (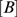) and RNA polymerase (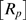) each can bind at different promoters in the triad when chromatin is on the open state with binding affinities that are represented here as free energies 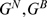 and 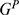 respectively. These free energies can vary for different promoters and also depend upon energy of interactions between different proteins bounds to DNA. We note that the triad enhancers bind TRs to regulate gene expression in a chromatin integration dependent manner [12]. Moreover the position of the enhancer does not affect its ability to regulate transcription. These results suggest that the enhancer bound TRs do not physically interact with promoter bound factors such as Notch, Bmp4 and RNA polymerase to affect transcription. Therefore we assume that the free energy of interaction between enhancer and promoter bound proteins is zero. We assume that the binding of Notch/Bmp4 and RNA polymerase at the promoter is cooperative. Under this assumption the binding of RNA polymerase at the promoters is enhanced by the free energies of its interaction with Notch (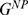) and Bmp4 (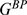). In our partition functions, we now account for configurations where either the enhancer or the promoter or both or neither are occupied by the various factors. We assume that 
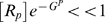
 because typical promoters bind RNA polymerase weakly and use this assumption to simplify the equations below.
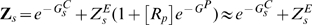
(12)

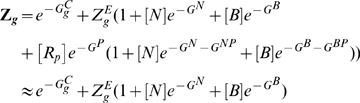
(13)

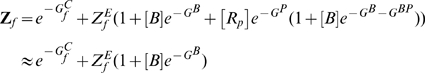
(14)Interestingly, even though we assumed in our derivation that there is no physical interaction between enhancer bound and promoter bound TRs we find that the partition functions of the *Gata2-3* and *Fli1+12* enhancers are not separable (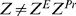) into distinct factors 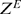 and 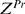 representing the partition functions for the enhancer states and promoter states respectively. Therefore the binding of TRs at the enhancers and the promoter is not independent. This emergence of cooperativity from competition of TRs with nucleosomes has been observed experimentally [50] and incorporated into mathematical models [9].

We define 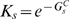, 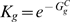 and 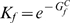 to be the equilibrium constants of chromatin rewrapping for the Scl, Gata and Fli1 respectively. Using equation (12), the probability of RNA polymerase being bound to the Scl promoter can be written as
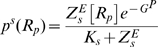
(15)Similarly we can write the expressions for the probability of Gata2 and Fli1 promoters being occupied by polymerases.
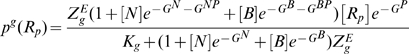
(16)

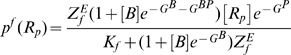
(17)We note that the effect of Notch and Bmp4 on the probability of transcription from the *Gata2-3* enhancer is saturable because Notch and Bmp4 concentrations (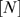 and 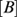 respectively) appear in both the numerator and denominator of the expression for 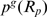 (cf. Eq. (16)). Similarly the effect of Bmp4 on the probability of transcription from the *Fli1+12* enhancer (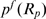) is also saturable (cf. Eq. (17)). The rate of gene expression for gene 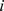, 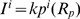 is assumed to be proportional to the probability of promoter occupation by RNA polymerase. The proportionality constant 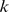 is the rate of isomerization of RNA polymerase to the open conformation. We rearrange the rate of gene expression as
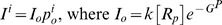
(18)

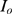
 represents the maximal rate of expression from the promoter in the open state. 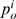 is a dimensionless rate of transcription that represents the cumulative regulatory effect of all enhancer and promoter bound TRs. Using equations (15)–(17) we can now write the expressions for 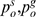 and 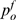.
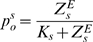
(19)

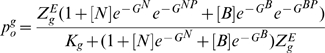
(20)

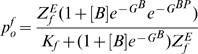
(21)


### Recursive estimation of free energies from experimental results

Deletion of binding sites from the enhancer 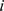 modifies 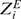, the partition coefficient for all bound configurations of that enhancer. Experimental results from the Gottgens group describe the fold-change in gene expression enhancement due to the selective mutation of certain enhancer binding sites [20]. Using their results for deletion of critical binding sites we can estimate the free energies of each TR-DNA interaction for the three enhancers. We use *Scl+19* as an illustrative example. Figure 1 shows the *Scl+19* enhancer and the fold expression enhancement for the reporter construct in the presence of the wildtype (*wt*) enhancer and three mutant enhancers: Mutant enhancer 1 (*mut1*)-Fli1 binding site deleted, Mutant enhancer 2 (*mut2*)-Gata2 binding site deleted, Mutant enhancer 3 (*mut3*)- all binding sites deleted. The transcription rates 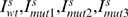 are normalized with the expression rate 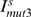 of the reporter when all enhancer binding sites have been deleted. We assume that the *lacZ* reporter transcription rates are proportional to the fluorescence intensities measured in these experiments because all experiments were performed in the presence of excess fluorescent substrate and wild-type and mutant constructs were assayed at the same time using the same reagents. Moreover the experimental conditions were controlled to ensure that the proportionality constants that relate various transcription rates 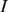 to the fluorescent intensities are the same for different experiments.

Note that the experimental results were obtained in HSCs which show high expression levels of Scl, Gata2 and Fli1 [20]. Notch and Bmp4 signals are expected to be absent in these cells [51]. We accordingly exclude all Notch and Bmp4 states from our partition functions. We can see from equations (19)–(21) that 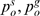 and 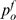 are the probabilities of the *Scl+19*, *Gata2-3* and *Fli1+12* enhancers being in open state in the absence of Notch and Bmp4.

The introduction of the mutant enhancer reporter construct is not expected to affect the growth rate or availability of RNA polymerases in a significant manner. Thus 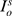 is unaffected by the deletion of binding sites. However the deletion of Fli1 binding sites eliminates the Fli1 bound state in the enhancer partition function 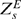 in equation (15). Therefore 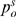 is affected by deletion of binding sites. Since 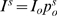, using equations (22)–(24) we can relate the fold enhancement in gene expression to the free energies of TR-DNA interaction.
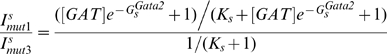
(22)

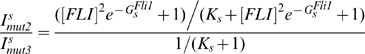
(23)

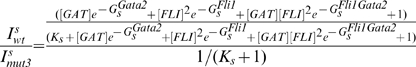
(24)Equations (22) and (23) can be solved analytically for 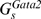 and 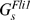 as functions of 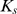 and the concentrations 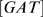 and 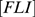.
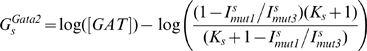
(25)

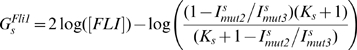
(26)The solution for 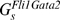 is dependent on 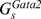 and 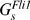. Using (25) and (26) we can solve for 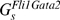 and reduce it to a function of only 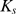, 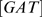 and 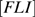.
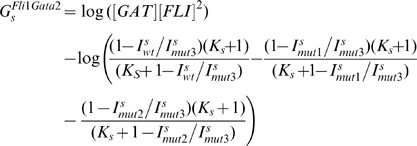
(27)We apply this recursive procedure to uniquely determine in a similar fashion all free energies of *Gata2-3* and *Fli1+12* enhancers. The full equations for all free energies are presented in Text S3 (see Eqs. (S.1)–(S.11)).

### Dynamical equations

Since mRNA is labile relative to stable cellular proteins, we assume that the mRNA concentration for the triad proteins is at steady state. We can thus directly relate the rate of transcription 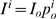 to the rate of production of the proteins
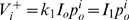
(28)(here 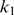 represents the number of protein molecules produced per mRNA lifetime). The ODEs for change in protein concentration can be written as a balance between the rate of production 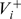 and the degradation/dilution rates that are linear in protein concentration (cf. Eq (4)).

The major hurdle in the analysis of this ODE model is the determination of all TR-enhancer interaction free energies from the equations described above (25)–(27) and in the supplement (S.1–S.11 in Text S3). The free energies can be determined from these relations if the concentrations of Scl, Gata2 and Fli1 in the wildtype cells and the constants 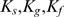 are known. However the TR concentrations are difficult to measure *in vivo*. We make our equations dimensionless to avoid the measurement of actual Scl, Gata2 and Fli1 concentrations. We normalize these TR concentrations by their wildtype concentrations. In wildtype HSCs the Scl, Gata2 and Fli1 concentrations are at steady state. Let these steady state wildtype concentrations be 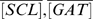 and 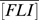. Normalizing Scl, Gata2 and Fli1 concentrations with 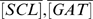 and 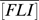 we can rewrite equation (4) as a system of ODEs in dimensionless variables 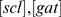 and 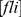 (cf. Eq (6)).

Rates 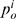 for all three enhancers as given by equations (19)–(21) can be recalculated in terms of the dimensionless variables by adjusting the free energies of each state with the appropriate concentrations. For example, 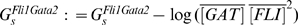. Note from equations (25)–(27) that the adjusted free energies are not functions of the steady state concentrations of Scl, Gata2 and Fli1.
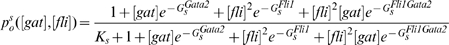


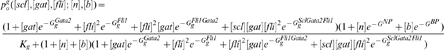


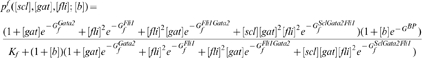
(29)Dimensionless rates 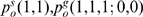 and 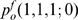 the wild-type, steady state dimensionless rates of transcription can be evaluated from the expressions in (29) by using adjusted free energies and 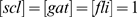. Then 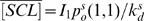, 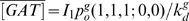 and 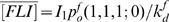.

The parameter space of free energies can now easily be explored by tuning 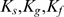. Since the free energies can be determined by fixing 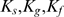, we can also analyze the system response to Notch and Bmp4 by substituting the full expressions of 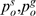 and 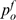 in equation (6).


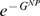
 and 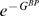 represent the strength of the interaction between RNA polymerase and Notch and Bmp4 respectively. Notch and Bmp4 increase Gata2 expression in wildtype HSCs by 3.5 [26] and 4 fold [27] respectively. At saturating concentrations of Notch (high 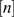) 
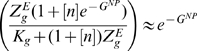
. This implies 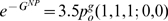. And similarly, 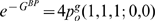. Thus 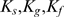 are the only unknown parameters in our model.

The model offers both a quantitative means of analysis of combinatorial regulation of gene expression by TRs and a succinct mathematical description of the biophysics of the regulation. The model can easily be extended to regulation involving repressors and many other situations.

The reduced model where Scl is not under regulation by Gata2 or Fli1 represents a simplification of this system where the concentration of Scl is kept constant. The reduced system then comprises only the equations for Gata2 and Fli1. The time normalization is carried out relative to the Scl half life (∼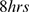) [43]. Gata2 and Fli1 have half-lives of ∼10 minutes and 2 hours respectively [44]. Accordingly 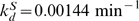, 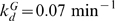 and 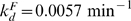. Our method for estimation of binding affinities reduces the number of unknown parameters in the system to three chromatin rewrapping equilibrium constants. These constants have been reported to be in the range 10–10000 [52]. We find that for irreversible bistable behavior with switchability our parameter estimation scheme restricts two of these equilibrium constants to a narrow range.
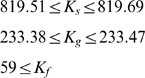
We chose the following values for the equilibrium constants: 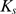 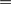 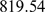, 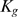 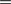 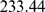 and 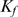 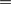 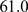 from within the ranges. The free energy values are thereafter calculated as described above to complete the parameter set for the triad model (cf. Table S3). The same parameter values are retained for the reduced model, however the Scl concentration in this case is fixed such that the threshold concentration (the concentration at the bifurcation point) of Notch/Bmp4 is identical for both the full triad and reduced model.

### Simulations

The system of equations for the triad described in the previous section was analyzed using a number of numerical methods and tools. The steady state characterization of both the triad and reduced modules was carried out using XPPAUT and the associated bifurcation analysis package AUTO [53]. Parameter sensitivity analysis for the chromatin equilibrium constants was also done with AUTO. The analysis of the dynamics of the ODE model was carried out using the ODE45 solver of MATLAB 2008a(R) (The MathWorks, Natick, Massachusetts). To compute the minimum pulse duration for Notch/Bmp4 signals, the integration was initiated at the low steady state and a step input of Notch/Bmp4 was introduced. The pulse duration to switch the system was minimized using the 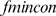 function (Optimization toolbox) in MATLAB. In all simulations only the dimensionless models were used.

## Supporting Information

Figure S1 Flow cytometry analysis of β-galactosidase expression from *Scl+19* enhancer-reporter constructs confirms all-or-none mechanism of gene regulation by distant enhancers.
(0.43 MB PDF)

Figure S2 Schematic diagram of ratchet model of distal enhancer action using *Scl+19* enhancer as an example.
(0.29 MB PDF)

Figure S3 Switchable bistability in triad response to Notch, Bmp4 and Gata1 is robust to variation in chromatin equilibrium constants.
(0.41 MB PDF)

Figure S4 Heterozygous deletions of *Scl*, *Gata2* and *Fli1* make the high expression state of the triad sensitive to fluctuations in TR levels.
(0.67 MB PDF)

Text S1 All or none regulation of gene expression from distant enhancers
(0.07 MB PDF)

Text S2 Modeling the effects of heterozygous deletions of triad genes
(0.06 MB PDF)

Text S3 Estimation of free energies from enhancer-reporter library expression results
(0.05 MB PDF)

Table S1 Enhancer-reporter library expression results
(0.04 MB PDF)

Table S2 Notation used in the main text and supplements
(0.03 MB PDF)

Table S3 Free energies for the triad enhancer-TR configurations
(0.04 MB PDF)
