# Supplementary material for: Modeling Reveals Bistability and Low-Pass Filtering in the Network Module Determining Blood Stem Cell Fate
Source: PLoS Comput Biol. 2010 May 6;6(5):e1000771. doi: 10.1371/journal.pcbi.1000771 (PMC2865510; doi:10.1371/journal.pcbi.1000771)
Supplement: Figure S1 — Flow cytometry analysis of β-galactosidase expression from Scl+19 enhancer-reporter constructs confirms all-or-none mechanism of gene regulation by distant enhancers. (0.43 MB PDF) [file pcbi.1000771.s001.pdf]

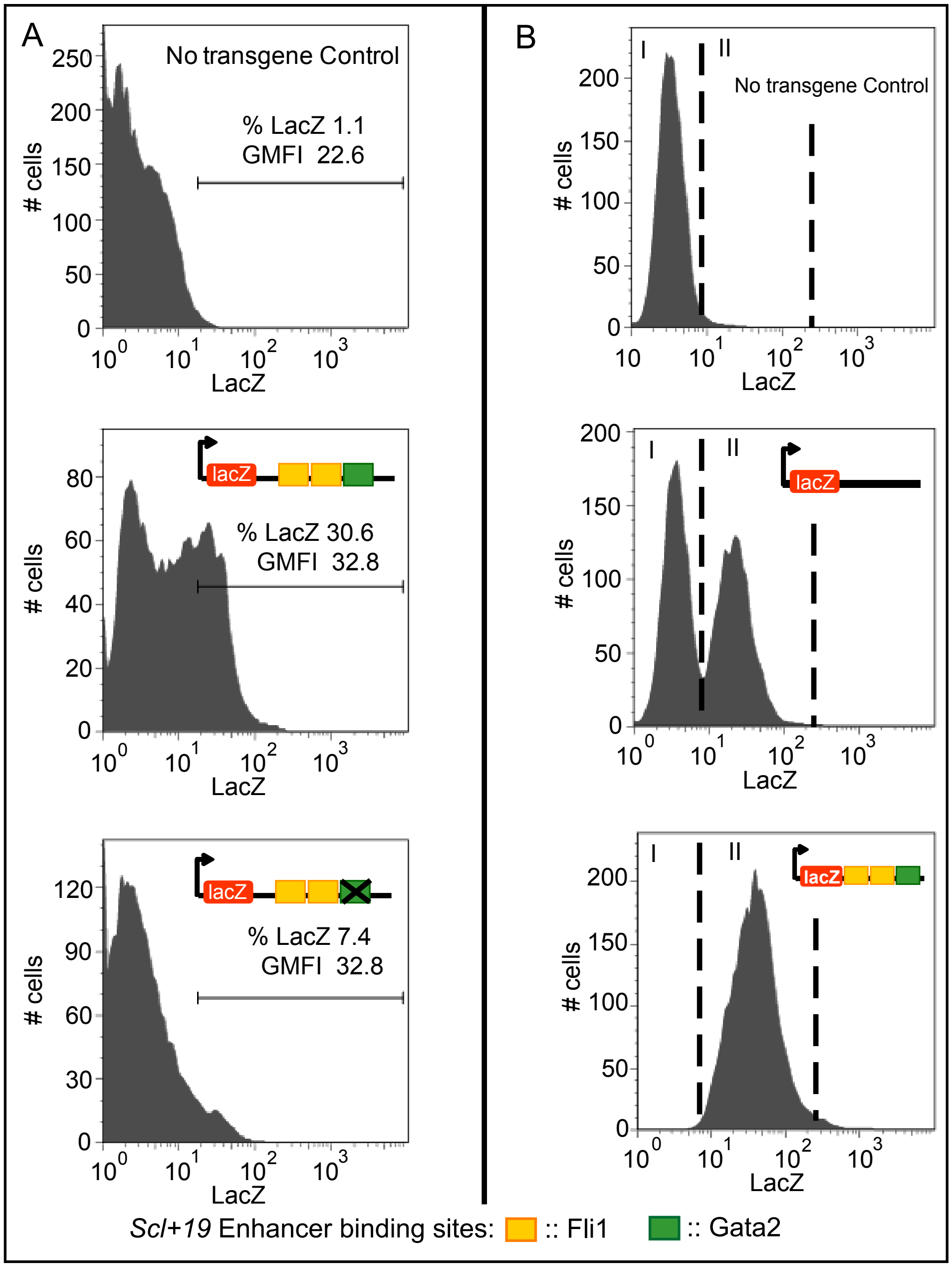


**Figure S1**. A. Flow cytometry analysis of β-galactosidase expression in day 5 embryoid bodies. A single copy of each of the reporter constructs for the *Scl +19* enhancer were targeted to the HPRT locus in embryonic stem (ES) cells. ES cells carrying either no transgene, the wild-type *Scl +19* enhancer or the *Scl +19* enhancer with the GATA site mutated were differentiated into embryoid bodies and analyzed at Day 5 of differentiation (onset of haematopoiesis) for the expression of β-galactosidase. The percentage of β-galactosidase positive cells is shown along-with the geometric mean fluorescence intensity (GMFI) of the β-galactosidase positive population. Note that the number of cells expressing β-galactosidase is reduced by the mutation in the enhancer but the mean level of expression in β-galactosidase positive cells is the same for both the wild type and the mutated enhancer. The ES cell data demonstrates a bi-modal distribution of β-galactosidase expression that remains in the presence of the mutated form of the enhancer showing that the *Scl+19* enhancer determines the number of cells expressing the enhancer but not the level of expression observed.

B. 416B myeloid progenitor cells were co-transfected with a puromycin expressing selection plasmid and either SV/β-geo or SV/β-geo/*Scl+19* reporter constructs. Pools of stably transfected cells were analysed for LacZ activity by flow cytometry. The dashed lines highlight the fact that the main consequence of including the enhancer is the loss of population I. By contrast, any increase in fluorescence in population II following inclusion of the enhancer is much less pronounced.
