# Supplementary material for: Modeling Reveals Bistability and Low-Pass Filtering in the Network Module Determining Blood Stem Cell Fate
Source: PLoS Comput Biol. 2010 May 6;6(5):e1000771. doi: 10.1371/journal.pcbi.1000771 (PMC2865510; doi:10.1371/journal.pcbi.1000771)
Supplement: Figure S2 — Schematic diagram of ratchet model of distal enhancer action using Scl+19 enhancer as an example. (0.29 MB PDF) [file pcbi.1000771.s002.pdf]

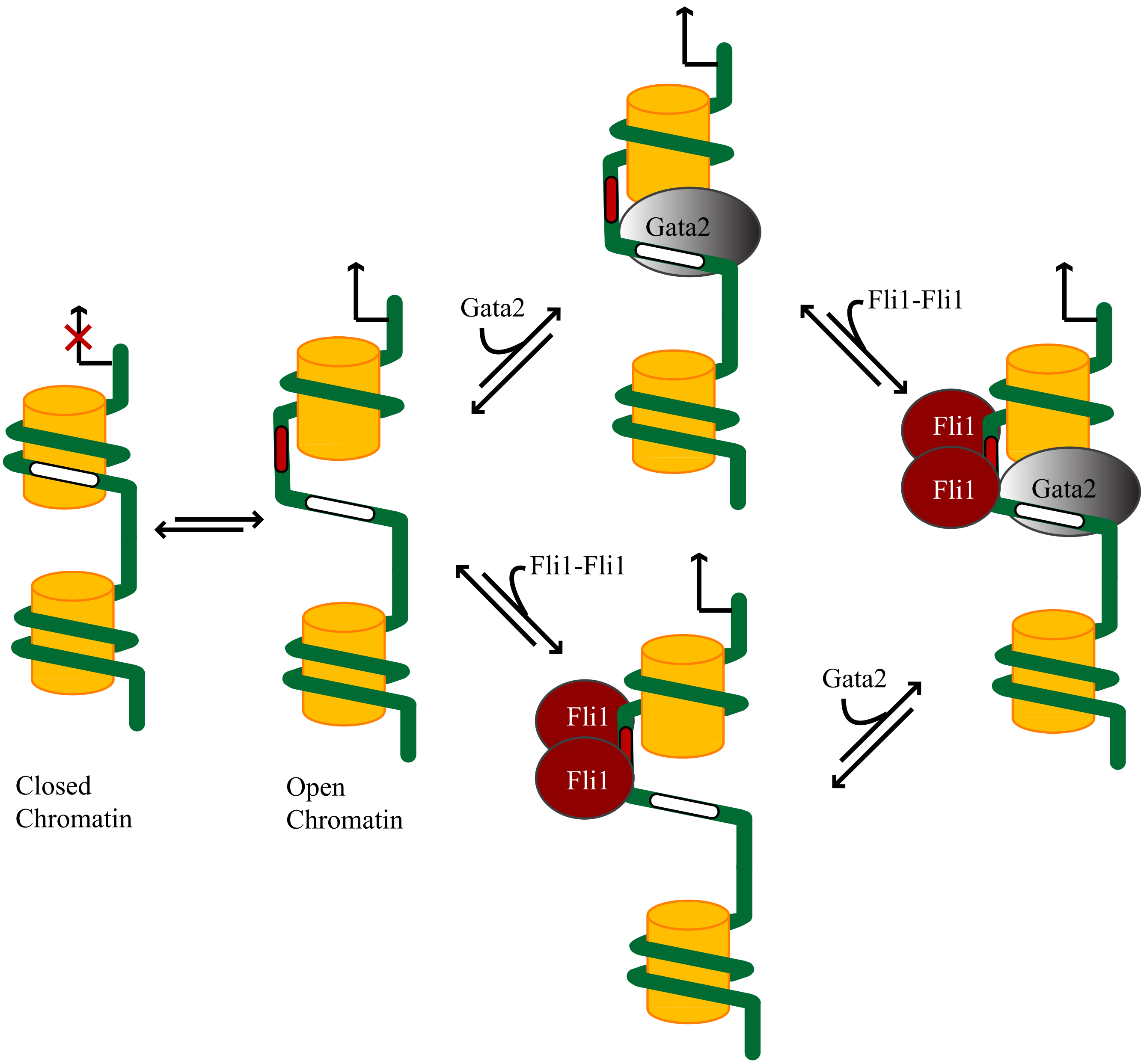

Figure S2. Schematic diagram of ratchet model of distal enhancer action using *Scl+19* enhancer as an example. The chromatin exists in an open and closed state as shown here. TRs and RNA polymerase can bind to DNA only in the open state. Gata2 and Fli1 bind to the enhancer and shift the equilibrium towards an open conformation. The increased probability of the open state results in increased gene expression.
