# Supplementary material for: Modeling Reveals Bistability and Low-Pass Filtering in the Network Module Determining Blood Stem Cell Fate
Source: PLoS Comput Biol. 2010 May 6;6(5):e1000771. doi: 10.1371/journal.pcbi.1000771 (PMC2865510; doi:10.1371/journal.pcbi.1000771)
Supplement: Figure S3 — Switchable bistability in triad response to Notch, Bmp4 and Gata1 is robust to variation in chromatin equilibrium constants. (0.41 MB PDF) [file pcbi.1000771.s003.pdf]

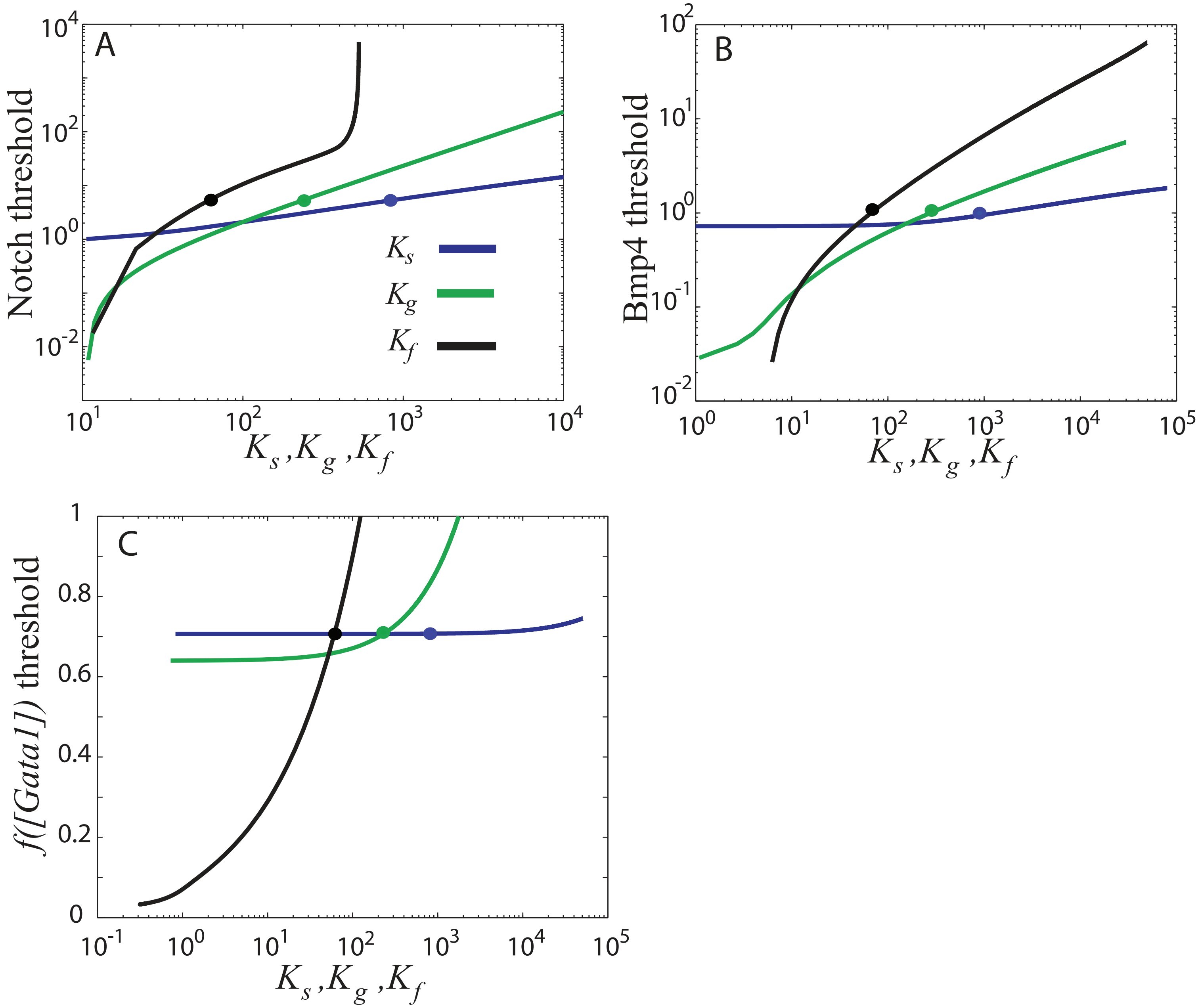

Figure S3. Robustness of switchable bistable response to variability in chromatin equilibrium constants. Switchable bistability in the triad response to Notch, Bmp4 and Gata1 is robust to large changes in chromatin equilibrium constants K_s, K_g and K_f when the free energies for the model are fixed at values given in Table S1. Threshold levels of Notch (A) and Bmp4 (B) for the *OFF-ON* switch and threshold levels of $f([Gata1])$ (C) for the deactivation switch *ON-OFF* change with the values of the chromatin equilibrium constants K_s (blue curve), K_g (green curve) and K_f (black curve). System response is bistable if threshold levels for Notch and Bmp4 are finite and lie within the range $0 < f([Gata1]) < 1$ for Gata1. Therefore the threshold levels indicate the ranges of chromatin equilibrium constant values where the system response is bistable. Moreover there is only one threshold for each signal over the range of parameter values shown here. Thus the irreversible nature of the bistable response is also robust to large changes in K_s, K_g and K_f. Solid dots indicate the values of K_s, K_g and K_f for which the dose response is given in Figures 2A and D.
