# Supplementary material for: Modeling Reveals Bistability and Low-Pass Filtering in the Network Module Determining Blood Stem Cell Fate
Source: PLoS Comput Biol. 2010 May 6;6(5):e1000771. doi: 10.1371/journal.pcbi.1000771 (PMC2865510; doi:10.1371/journal.pcbi.1000771)
Supplement: Figure S4 — Heterozygous deletions of Scl, Gata2 and Fli1 make the high expression state of the triad sensitive to fluctuations in TR levels. (0.67 MB PDF) [file pcbi.1000771.s004.pdf]

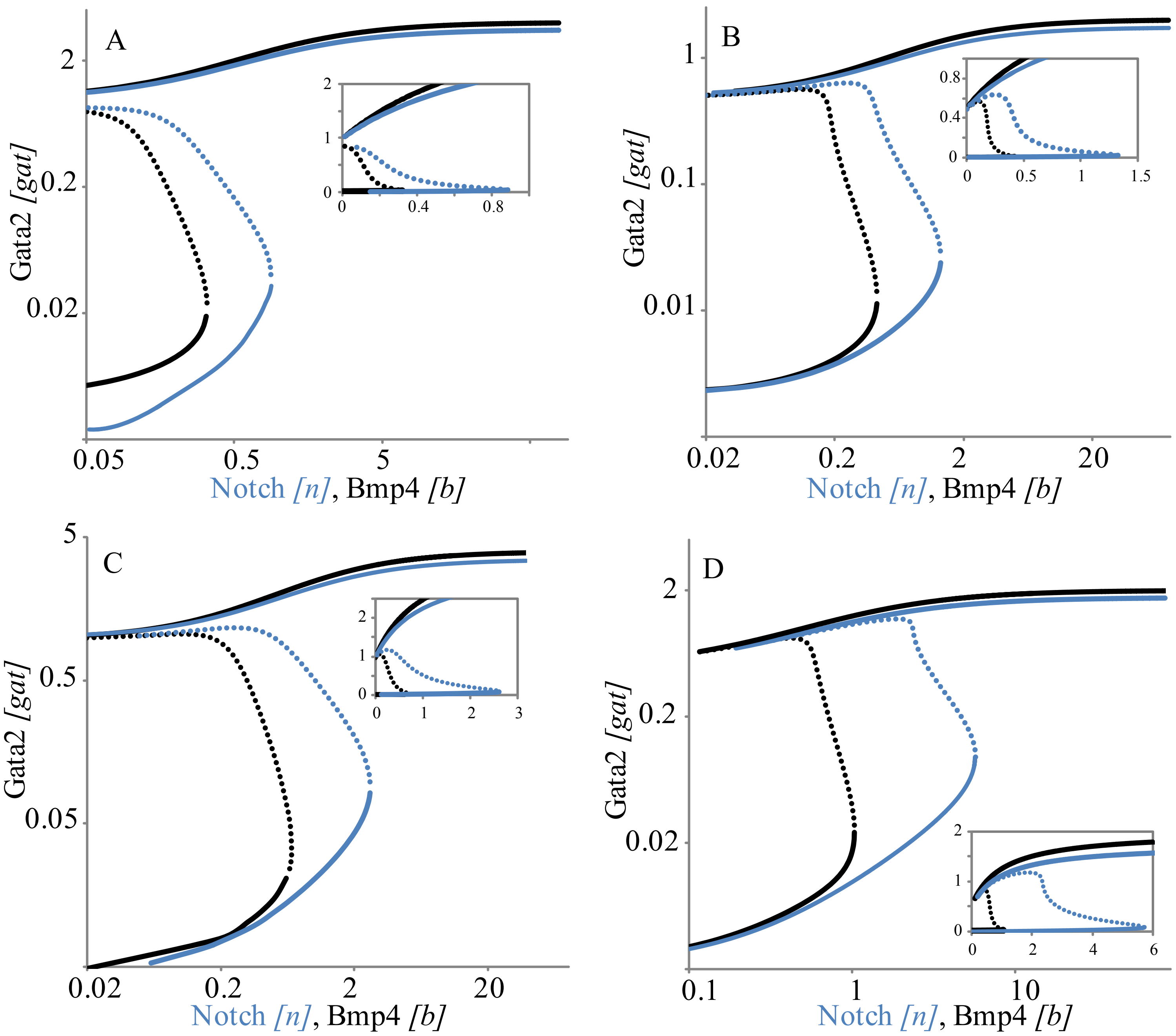

Figure S4. Dose-response to Notch and Bmp4 in Gata2, Scl and Fli1 heterozygotes. Scl+/- (A), Gata2+/- (B) and Fli1+/- (C) all show an irreversible bistable response to Notch (blue) and Bmp4 (black). Stable and unstable states are denoted by solid lines and dashed lines respectively. (D) Heterozygous deletion of two genes Gata2+/- Fli1+/- (results in reversible bistability in response to both Notch and Bmp4, so that at low levels of Notch and Bmp4, only the low steady state of triad proteins exists. The insets depict the dose response in linear scale to show that all heterozygous mutants except Gata2+/- and Fli1+/- have two steady states in the absence of Notch and Bmp4. Note that in B-D the basin of attraction for the high steady state is very small compared to the wild type (Figure 2A) for low values of activators, especially Notch. The high steady state of heterozygous deletions can therefore be very sensitive to fluctuations in protein concentrations in the absence of Notch and Bmp4.
