# Supplementary material for: Modeling Reveals Bistability and Low-Pass Filtering in the Network Module Determining Blood Stem Cell Fate
Source: PLoS Comput Biol. 2010 May 6;6(5):e1000771. doi: 10.1371/journal.pcbi.1000771 (PMC2865510; doi:10.1371/journal.pcbi.1000771)
Supplement: Text S1 — All or none regulation of gene expression from distant enhancers (0.07 MB PDF) [file pcbi.1000771.s005.pdf]

Text S1

All or none regulation of gene expression from distant enhancers

We propose that the Scl, Gata2 and Fli1 regulate gene expression by ratcheting the equilibrium between open (promoter site accessible) and closed (promoter site inaccessible) chromatin conformations. This dynamic equilibrium of wrapped and unwrapped nucleosomal DNA has also been discussed elsewhere [1]. Under our hypothesis the binding of TRs to the enhancer site increases gene expression by stabilizing the open conformation and thereby shifting the equilibrium towards it. This mechanism therefore allows the TRs to ratchet the spontaneous unwrapping of nucleosomal DNA and trap it in a state accessible to the transcriptional machinery. We illustrate this mechanism of the regulation of *Scl* expression by Gata2 and Fli1 in Figure S2. According to this mechanism binding of TRs only modulates the probability of gene expression and not the rate of transcription. Therefore, mutations of binding sites in the enhancers would affect the number of cells expressing the gene but the level of expression would be unaffected. To experimentally verify this all-or-none type of gene regulation embryonic stem cells containing the *Scl+19* enhancer-reporter constructs in Figure 1 were analyzed for reporter expression with flow cytometry (Figure S1A). Mutation of the binding sites significantly reduces the fraction of cells expressing β-galactosidase. However the mean β-galactosidase expression among cells that do show expression did not change thereby confirming our hypothesis about the mechanism of gene regulation by TR binding at distant enhancers. The bi-modality of the data is obscured by the presence of several different cell types in the culture of ES cells. To highlight the all-or-none nature of gene regulation by the triad enhancers a similar experiment was carried out in 416B myeloid progenitor cells that were transfected with either SV/β-geo or SV/β-geo/*Scl+19* reporter constructs. Figure S2B shows that the inclusion of the *Scl+19* enhancer significantly enhances the number of cells expressing lacZ while the level of lacZ expression is unaffected. These results confirm our hypothesis about the all-or-none nature of gene regulation by enhancers within the Scl/Gata2/Fli1 triad.

Experimental Methods

The wild type and mutated *Scl+19* enhancer elements previously described in Pimanda et al [2] and shown in Figure 1 were cloned downstream of an SV40LacZ reporter construct and inserted as a Not I blunt fragment into the HPRT targeting vector pMP8NEBΔLacZ (a kind gift from Stephen Duncan) [3]. HPRT targeted ES cells were generated as previously described [4] using the HM-1 ES cell line [5] and differentiated in 90mm Petri dishes in IMDM supplemented with 15% FCS, 2mM L-Glutamine, 300ug/ml Transferrin, 4x10-4M MTG, 50ug/ml Ascorbic acid and 5% PFHM-II. At day 5 of differentiation embryoid bodies were disrupted using trypsin and the single-cell suspension was analysed for β-galactosidase activity using FDG (fluorescein di-β-D-galactoside) [6]. Briefly, cells were incubated with 1mM FDG at 37°C for 1 min followed by the addition of 1ml ice cold PBS. The cells were then incubated on ice for 10 mins and the

reaction stopped using 1mM phenylethyl-β-D-thiogalactoside. The fluorescent signal was then analysed on the FACS Calibur (Becton and Dickinson) in the FL-1 channel. The *Gata2-3* and *Fli1+12* enhancers have been characterized in [2]. The results for reporter expression from wild type and mutated enhancer elements for all three triad enhancers are shown in Table S1.

1. Polach KJ, Widom J (1995) Mechanism of protein access to specific DNA sequences in chromatin: a dynamic equilibrium model for gene regulation. J Mol Biol 254(2): 130-49.
2. Pimanda JE, Ottersbach K, Knezevic K, Kinston S, Chan WY, et al. (2007) Gata2, Fli1, and Scl form a recursively wired gene-regulatory circuit during early hematopoietic development. Proc Natl Acad Sci U S A 104(45): 17692-7.
3. Misra RP, Bronson SK, Xiao Q, Garrison W, Li J, et al. (2001) Generation of single-copy transgenic mouse embryos directly from ES cells by tetraploid embryo complementation. BMC Biotechnol 1: 12.
4. Smith AM, Sanchez MJ, Follows GA, Kinston S, Donaldson IJ, et al. (2008) A novel mode of enhancer evolution: the Tal1 stem cell enhancer recruited a MIR element to specifically boost its activity. Genome Res 18(9): 1422-32.
5. Selfridge J, Pow AM, McWhir J, Magin TM, Melton DW (1992) Gene targeting using a mouse HPRT minigene/HPRT-deficient embryonic stem cell system: inactivation of the mouse ERCC-1 gene. Somat Cell Mol Genet 18(4): 325-36.
6. Fiering SN, Roederer M, Nolan GP, Micklem DR, Parks DR, et al. (1991) Improved FACS-Gal: flow cytometric analysis and sorting of viable eukaryotic cells expressing reporter gene constructs. Cytometry 12(4): 291-301.
