# Supplementary material for: Modeling Reveals Bistability and Low-Pass Filtering in the Network Module Determining Blood Stem Cell Fate
Source: PLoS Comput Biol. 2010 May 6;6(5):e1000771. doi: 10.1371/journal.pcbi.1000771 (PMC2865510; doi:10.1371/journal.pcbi.1000771)
Supplement: Text S2 — Modeling the effects of heterozygous deletions of triad genes (0.06 MB PDF) [file pcbi.1000771.s006.pdf]

Text S2

Modeling the effects of heterozygous deletions of triad genes

HSCs heterozygous for triad proteins have been shown to have defects in repopulation capacity [1, 2]. We use our model to analyze the effect of heterozygous deletions of triad genes on triad response to Notch and Bmp4. *Scl+/-*, *Gata2+/-* and *Fli1+/-* heterozygotes all show bistable dose response to Notch and Bmp4 (Figure S4 A-C). Note that the basins of attraction for the high steady states of heterozygous mutants at low concentration of Notch/Bmp4 are very small. These mutants should therefore be sensitive to stochastic fluctuations in triad protein concentrations. We expect that the noise induced switching from *ON* to *OFF* state in these mutants would be much higher than that for wild type cells. If the triad is a master regulator of the maintenance of stem cell state an increased rate of switching from *ON* to *OFF* state would result in an increase in the fraction of stem cells that produce multipotent progenitor cells committed to differentiation and a decrease in stem cell renewal. The decreased rate of stem cell proliferation would explain the deficient repopulation capacity of *Scl+/-*, *Gata2+/-* and *Fli1+/-* heterozygotic mutants [1, 2]. We also analyzed the effects of heterozygous deletion of both the Gata2 and Fli1 genes and find that this cell line can only display reversible bistability-switching back to *OFF* state at low Notch and Bmp4 concentrations. Therefore, we predict a depletion of HSC pool in this mutant.

1. Curtis DJ, Hall MA, Van Stekelenburg LJ, Robb L, Jane SM, et al. (2004) SCL is required for normal function of short-term repopulating hematopoietic stem cells. Blood 103(9): 3342-8.
2. Rodrigues NP, Janzen V, Forkert R, Dombkowski DM, Boyd AS, et al. (2005) Haploinsufficiency of GATA-2 perturbs adult hematopoietic stem-cell homeostasis. Blood 106(2): 477-84.
