# Supplementary material for: Modeling Reveals Bistability and Low-Pass Filtering in the Network Module Determining Blood Stem Cell Fate
Source: PLoS Comput Biol. 2010 May 6;6(5):e1000771. doi: 10.1371/journal.pcbi.1000771 (PMC2865510; doi:10.1371/journal.pcbi.1000771)
Supplement: Text S3 — Estimation of free energies from enhancer-reporter library expression results (0.05 MB PDF) [file pcbi.1000771.s007.pdf]

Text S3

Estimation of free energies from enhancer library expression results

Our approach for the calculation of free energies of TR-DNA and TR-TR interactions is discussed in Methods. The thermodynamic model is used to match the experimental results for the fold enhancement of gene expression from each enhancer (see Table S1 for experimental results). The experimental results are then used to reduce the dimension of the parameter space of the model. The following equations were derived to calculate the free energies that are parameters in the model from values of the chromatin equilibrium constants and the experimentally observed fold enhancement in expression.

Free energies for *Scl*+19:

$$\text{Gata2 binding: } G_s^{Gata2} = \log([GAT]) - \log\left(\frac{(1 - I_{mut1}^s / I_{mut3}^s)(K_s + 1)}{(K_s + 1 - I_{mut1}^s / I_{mut3}^s)}\right) \tag{S.1}$$

$$\text{Fli1 binding: } G_s^{Fli1} = 2\log([FLI]) - \log\left(\frac{(1 - I_{mut2}^s / I_{mut3}^s)(K_s + 1)}{(K_s + 1 - I_{mut2}^s / I_{mut3}^s)}\right) \tag{S.2}$$

Gata2-Fli1 binding:

$$G_s^{Fli1Gata2} = \log([GAT][FLI]^2) - \log\left(\frac{(1 - I_{wt}^s / I_{mut3}^s)(K_s + 1)}{(K_s + 1 - I_{wt}^s / I_{mut3}^s)} - \frac{(1 - I_{mut1}^s / I_{mut3}^s)(K_s + 1)}{(K_s + 1 - I_{mut1}^s / I_{mut3}^s)} - \frac{(1 - I_{mut2}^s / I_{mut3}^s)(K_s + 1)}{(K_s + 1 - I_{mut2}^s / I_{mut3}^s)}\right) \tag{S.3}$$

Free energies for *Gata2-3*:

$$\text{Gata2 binding: } G_g^{Gata2} = \log([GAT]) - \log\left(\frac{(1 - I_{mut1}^g / I_{mut3}^g)(K_g + 1)}{(K_g + 1 - I_{mut1}^g / I_{mut3}^g)}\right) \tag{S.4}$$

$$\text{Fli1 binding: } G_g^{Fli1} = 2\log([FLI]) - \log\left(\frac{(1 - I_{mut2}^g / I_{mut3}^g)(K_g + 1)}{(K_g + 1 - I_{mut2}^g / I_{mut3}^g)}\right) \tag{S.5}$$

Gata2-Fli1 binding:

$$G_g^{Fli1Gata2} = \log([GAT][FLI]^2) - \log\left(\frac{(1 - I_{wt}^g / I_{mut3}^g)(K_g + 1)}{(K_g + 1 - I_{wt}^g / I_{mut3}^g)} - \frac{(1 - I_{mut1}^g / I_{mut3}^g)(K_g + 1)}{(K_g + 1 - I_{mut1}^g / I_{mut3}^g)} - \frac{(1 - I_{mut2}^g / I_{mut3}^g)(K_g + 1)}{(K_g + 1 - I_{mut2}^g / I_{mut3}^g)}\right) \tag{S.6}$$

Scl-Gata2-Fli1 binding:

$$G_g^{SclGata2Fli1} = \log([SCL][GAT][FLI]^2) - \log\left(\frac{(1 - I_{wt}^g / I_{mut4}^g)(K_g + 1)}{(K_g + 1 - I_{wt}^g / I_{mut4}^g)} - \frac{(1 - I_{mut3}^g / I_{mut4}^g)(K_g + 1)}{(K_g + 1 - I_{mut3}^g / I_{mut4}^g)} \right) \quad \text{(S.7)}$$

Free energies for *Fli1+12*:

$$\text{Gata2 binding: } G_f^{Gata2} = \log([GAT]) - \log\left(\frac{(1 - I_{mut1}^f / I_{mut3}^f)(K_f + 1)}{(K_f + 1 - I_{mut1}^f / I_{mut3}^f)} \right) \quad \text{(S.8)}$$

$$\text{Fli1 binding: } G_f^{Fli1} = 2\log([FLI]) - \log\left(\frac{(1 - I_{mut2}^f / I_{mut3}^f)(K_f + 1)}{(K_f + 1 - I_{mut2}^f / I_{mut3}^f)} \right) \quad \text{(S.9)}$$

Gata2-Fli1 binding:

$$G_f^{Fli1Gata2} = \log([GAT][FLI]^2) - \log\left(\frac{(1 - I_{wt}^f / I_{mut3}^f)(K_f + 1)}{(K_f + 1 - I_{wt}^f / I_{mut3}^f)} - \frac{(1 - I_{mut1}^f / I_{mut3}^f)(K_f + 1)}{(K_f + 1 - I_{mut1}^f / I_{mut3}^f)} - \frac{(1 - I_{mut2}^f / I_{mut3}^f)(K_f + 1)}{(K_f + 1 - I_{mut2}^f / I_{mut3}^f)} \right) \quad \text{(S.10)}$$

Scl-Gata2-Fli1 binding:

$$G_f^{SclGata2Fli1} = \log([SCL][GAT]^2[FLI]^2) - \log\left(\frac{(1 - I_{wt}^f / I_{mut4}^f)(K_f + 1)}{(K_f + 1 - I_{wt}^f / I_{mut4}^f)} - \frac{(1 - I_{mut3}^f / I_{mut4}^f)(K_f + 1)}{(K_f + 1 - I_{mut3}^f / I_{mut4}^f)} \right) \quad \text{(S.11)}$$

All free energies can be determined using the values of chromatin rewrapping equilibrium constants in these equations. The dimensions of the parameter space are therefore reduced to just these equilibrium constants. We choose K_s, K_g and K_f from a range of values where the triad exhibits switchable bistability and determine free energies for the chosen set of parameter values. The free energies are shown in Table S3. The free energy of TR-DNA binding varies between enhancers due to the effect of sequences flanking the binding sites. Notably the TR-TR interaction (e.g. Gata2-Fli1) energies vary very little between enhancers.
