# Supplementary material for: Modeling Reveals Bistability and Low-Pass Filtering in the Network Module Determining Blood Stem Cell Fate
Source: PLoS Comput Biol. 2010 May 6;6(5):e1000771. doi: 10.1371/journal.pcbi.1000771 (PMC2865510; doi:10.1371/journal.pcbi.1000771)
Supplement: Table S1 — Enhancer-reporter library expression results (0.04 MB PDF) [file pcbi.1000771.s008.pdf]

Table S1

Table S1. Enhancer library reporter expression results (adapted from [1])

	Enhancers	Binding Sites	Fold Enhancement of Expression Rate *I*
Scl+19	Wild Type (wt)	Ets-Ets-GATA	820.51
	Mutant 1 (mut1)	GATA	141.02
	Mutant 1 (mut2)	Ets-Ets	235.89
	Mutant 1 (mut3)	-	1
Gata2-3	Wild Type (wt)	EBOX-GATA-Ets-Ets	234.38
	Mutant 1 (mut1)	GATA	1.72
	Mutant 2 (mut2)	Ets-Ets	1.72
	Mutant 3 (mut3)	GATA-Ets-Ets	120.31
	Mutant 4 (mut4)	-	1
Fli1+12	Wild Type (wt)	GATA-EBOX-Ets-Ets-GATA	60
	Mutant 1 (mut1)	GATA	1.5
	Mutant 2 (mut2)	Ets-Ets	1.5
	Mutant 3 (mut3)	GATA-Ets-Ets	20
	Mutant 4 (mut4)	-	1

1. Pimanda JE, Ottersbach K, Knezevic K, Kinston S, Chan WY, et al. (2007) Gata2, Fli1, and Scl form a recursively wired gene-regulatory circuit during early hematopoietic development. Proc Natl Acad Sci U S A 104(45): 17692-7.
