# Supplementary material for: Modeling Reveals Bistability and Low-Pass Filtering in the Network Module Determining Blood Stem Cell Fate
Source: PLoS Comput Biol. 2010 May 6;6(5):e1000771. doi: 10.1371/journal.pcbi.1000771 (PMC2865510; doi:10.1371/journal.pcbi.1000771)
Supplement: Table S2 — Notation used in the main text and supplements (0.03 MB PDF) [file pcbi.1000771.s009.pdf]

Table S2. Notation for various symbols used in the main text and supplementary information

	Symbol	Name
Free Energies of different configurations	G_i^C	Closed chromatin conformation of enhancer *i*: (*i = s* (*Scl+19*), *g (Gata2-3), f (Fli1+12)*)
	G_i^{Gata2}	Gata2 bound to enhancer *i*: (*i = s* (*Scl+19*), *g (Gata2-3), f (Fli1+12)*)
	G_i^{Fli1}	Fli1 bound to enhancer *i*: (*i = s* (*Scl+19*), *g (Gata2-3), f (Fli1+12)*)
	$G_i^{Fli1Gata2}$	Gata2& Fli1 bound to enhancer *i*: (*i = s* (*Scl+19*), *g (Gata2-3), f (Fli1+12)*)
	$G_i^{SclGata2Fli1}$	Scl&Gata2&Fli1 bound to enhancer *i*: (*i = g (Gata2-3), f (Fli1+12)*)
	G^N	Notch bound to promoter
	G^B	Bmp4 bound to promoter
	G^P	Polymerase bound to promoter
Free energies of interaction	G^{BP}	Bmp4 and RNA polymerase interaction energy
	G^{NP}	Notch and RNA polymerase interaction energy
Chromatin Equilibrium Constants	K_i	Chromatin equilibrium constant for enhancer *i*: (*i = s* (*Scl+19*), *g (Gata2-3), f (Fli1+12)*) $K_i = e^{-G_i^C}$
Concentrations	*[SCL]*	Intracellular Scl concentration
	[GAT]	Intracellular Gata2 concentration
	[FLI]	Intracellular Fli1 concentration
	[Gata1]	Intracellular Gata1 concentration
	[N]	Intracellular Notch concentration
	[B]	Intracellular Bmp4 concentration
	$[R_p]$	Intracellular RNA polymerase concentration
Steady State Concentrations	$\left[\overline{SCL}\right]$	Scl concentration in high steady state
	$\left[\overline{GAT}\right]$	Gata2 concentration in high steady state
	$\left[\overline{FLI}\right]$	Fli1 concentration in high steady state

Dimensionless Concentrations	$\left[scl\right]$	Concentration of Scl relative to concentration in high steady state. $\left[scl\right]=\left[SCL\right]/\overline{\left[SCL\right]}$
	$\left[gat\right]$	Concentration of Gata2 relative to concentration in high steady state. $\left[gat\right]=\left[GAT\right]/\overline{\left[GAT\right]}$
	$\left[fli\right]$	Concentration of Fli1 relative to concentration in high steady state. $\left[fli\right]=\left[FLI\right]/\overline{\left[FLI\right]}$
	$\left[n\right]$	Concentration of Notch relative to dissociation constant of binding to the promoter. $\left[n\right]=\left[N\right]/e^{G^N}$
	$\left[b\right]$	Concentration of Scl relative to concentration in high steady state. $\left[b\right]=\left[B\right]/e^{G^B}$
Other symbols used	p_o^i	Probability of open chromatin conformation for enhancer *i*: (*i = s (Scl+19), g (Gata2-3), f (Fli1+12))*
	$p^i(R_p)$	Probability of RNA polymerase being bound to promoter for enhancer-reporter construct with enhancer *i*: (*i = s (Scl+19), g (Gata2-3), f (Fli1+12)).* $p^i(R_p)=\left[R_p\right]e^{-G^P}p_o^i$
	I_o^i	Maximum rate of transcription from SV promoter. $I_o=kR_pe^{-G^P}$
	I^i	Rate of transcription from SV promoter-enhancer construct for enhancer *i*: (*i = s (Scl+19), g (Gata2-3), f (Fli1+12))* $I^i=I_op_o^i$
	I_1	Maximum rate of protein production from SV promoter. $I_1=k_1I_o$
	k	Rate of isomerization of SV promoter bound RNA polymerase to open conformation
	k_1	Number of proteins produced per lifetime of mRNA
	$\mathbf{Z_i}$	Partition function for enhancer *i*: (*i = s (Scl+19), g (Gata2-3), f (Fli1+12))*
	Z_i^E	Sum of Boltzmann weights for all open chromatin state enhancer configurations of enhancer *i*. (*i*=*s* (*Scl+19*), *g* (*Gata2-3*), *f* (*Fli1+12*)).
	k_d^i	Degradation rate for TR *i*: (*i*=*s* (Scl), *g* (Gata2), *f* (Fli1)).
