# Supplementary material for: Modeling Reveals Bistability and Low-Pass Filtering in the Network Module Determining Blood Stem Cell Fate
Source: PLoS Comput Biol. 2010 May 6;6(5):e1000771. doi: 10.1371/journal.pcbi.1000771 (PMC2865510; doi:10.1371/journal.pcbi.1000771)
Supplement: Table S3 — Free energies for the triad enhancer-TR configurations (0.04 MB PDF) [file pcbi.1000771.s010.pdf]

Table S3

Table S3. Free energy values used in the model for Scl-Gata2-Fli1 triad

	TR	Symbol	Value
Scl+19	Gata2	G_s^{Gata2}	-5.1304
	Fli1	G_s^{Fli1}	-2.8991
	Fli1-Gata2	$G_s^{Fli1Gata2}$	-17.0239
Gata2-3	Gata2	G_g^{Gata2}	0.3229
	Fli1	Gg^{Fli1}	0.1614
	Fli1-Gata2	$G_g^{Fli1Gata2}$	-5.4957
	Scl-Fli1-Gata2	$G_g^{SclGata2Fli1}$	-13.6429
Fli1+12	Gata2	G_f^{Gata2}	0.6687
	Fli1	G_f^{Fli1}	0.3343
	Fli1-Gata2	$G_f^{Fli1Gata2}$	-3.2967
	Scl-Gata2-Fli1	$G_f^{SclGata2Fli1}$	-7.4961
